# Phenylethanoid and Phenylmethanoid Glycosides from the Leaves of *Ligustrum robustum* and Their Bioactivities

**DOI:** 10.3390/molecules27217390

**Published:** 2022-10-31

**Authors:** Shi-Hui Lu, Hao-Jiang Zuo, Jing Huang, Ran Chen, Jia-Ping Pan, Xiu-Xia Li

**Affiliations:** 1 College of Pharmacy, Youjiang Medical University for Nationalities, Baise 533000, China; 2 Department of Laboratory Science of Public Health, West China School of Public Health, Sichuan University, Chengdu 610041, China; 3 Key Laboratory of Drug Targeting, Ministry of Education, West China School of Pharmacy, Sichuan University, Chengdu 610041, China; 4 Institute of Life Science, Youjiang Medical University for Nationalities, Baise 533000, China; 5 Nursing School, Youjiang Medical University for Nationalities, Baise 533000, China

**Keywords:** *Ligustrum robustum*, phenylethanoid glycoside, phenylmethanoid glycosides, FAS, *α*-glucosidase, antioxidant, anti-obesity, hypoglycemic

## Abstract

The phytochemical study on the leaves of *Ligustrum robustum*, which have been used as Ku-Ding-Cha, led to the isolation and identification of three new phenylethanoid glycosides and three new phenylmethanoid glycosides, named ligurobustosides R_1_ (**1b**), R_2–3_ (**2**), R_4_ (**3**), S_1_ (**4b**), S_2_ (**5**), and S_3_ (**6**), and five reported phenylethanoid glycosides (**7**–**11**). In the bioactivity test, (*Z*)-osmanthuside B_6_ (**11**) displayed strong fatty acid synthase (FAS) inhibitory activity (IC_50_: 4.55 ± 0.35 μM) as the positive control orlistat (IC_50_: 4.46 ± 0.13 μM), while ligurobustosides R_4_ (**3**) and S_2_ (**5**), ligupurpuroside B (**7**), *cis*-ligupurpuroside B (**8**), ligurobustoside N (**9**), osmanthuside D (**10**), and (*Z*)-osmanthuside B_6_ (**11**) showed stronger ABTS radical scavenging activity (IC_50_: 2.68 ± 0.05~4.86 ± 0.06 μM) than the positive control L-(+)-ascorbic acid (IC_50_: 10.06 ± 0.19 μM). This research provided a theoretical basis for the leaves of *L. robustum* as a tea with function in treating obesity and diabetes.

## 1. Introduction

**Figure 1:**
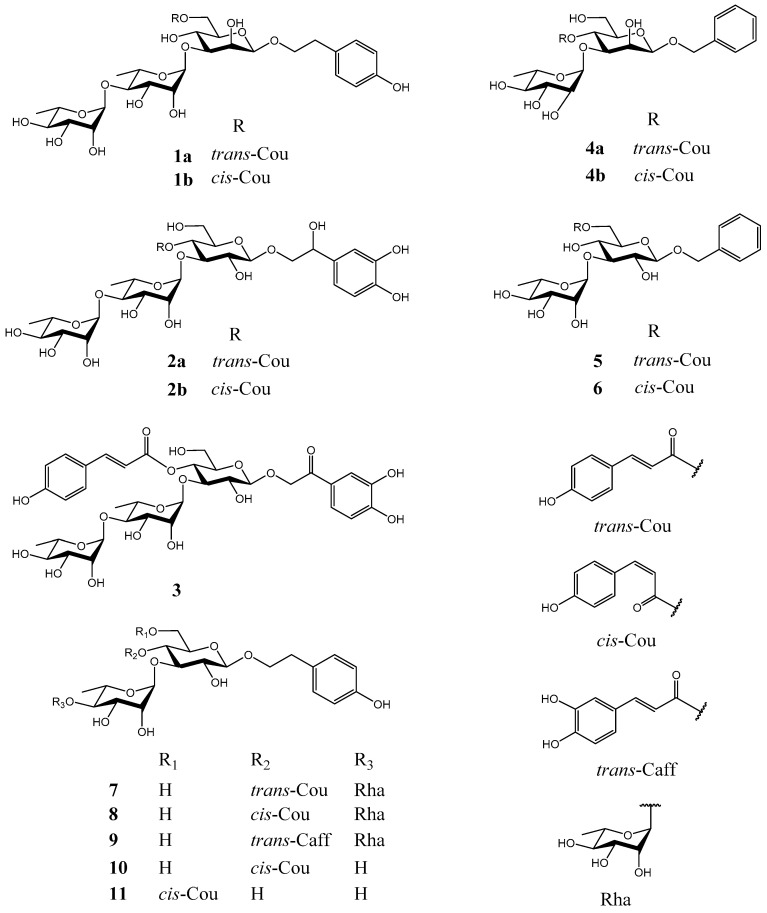
Structures of compounds **1**–**11** from the leaves of *L. robustum*.

Ku-Ding-Cha, a tea with functions in clearing heat, removing toxins, and treating obesity and diabetes, has been applied widely in Southwest China for nearly 2000 years [1,2]. It was derived from the leaves of more than 30 plants belonging to 13 genera in 12 families [3]. *Ligustrum robustum* (Roxb.) Blume (Oleaceae), classified as a food by the Chinese Ministry of Health since 2011, has been used as Ku-Ding-Cha in Southwest China [4,5]. In the previous investigations on *L.* *robustum* [1,2,3,4,5,6,7,8,9,10,11,12,13,14,15,16], more than 60 chemical constituents, including monoterpenoid glycosides, phenylethanoid glycosides, phenylmethanoid glycosides, iridoid glycosides, flavonoid glycosides, lignan glycosides, and triterpenoids, were discovered, and the antioxidative, anti-obesity, and anti-inflammatory effects of the aqueous extract, the inhibitory activities on FAS, *α*-glucosidase, and *α*-amylase, and the antioxidant effects of some constituents, were observed. To further elucidate the active components for preventing obesity and diabetes, the phytochemical and biological study on the leaves of *L.* *robustum*, which had been performed preliminarily [12,13], was carried out. As a result, three new phenylethanoid glycosides and three new phenylmethanoid glycosides, named ligurobustosides R_1_ (**1b**), R_2–3_ (**2**), R_4_ (**3**), S_1_ (**4b**), S_2_ (**5**), and S_3_ (**6**), and five reported phenylethanoid glycosides (**7**–**11**) (Figure 1) were isolated from the leaves of *L. robustum*. This article discusses the isolation and structure identification of compounds **1**–**11** and deals with their inhibitory effects on FAS, *α*-glucosidase, *α*-amylase, and their antioxidant activities.

## 2. Material and Methods

### 2.1. General Experimental Procedure

Optical rotation value was determined with an AUTOPOL VI automatic polarimeter (Rudolph, Hackettstown, NJ, USA). The UV spectrum was measured on a UV2700 spectrophotometer (Shimadzu, Kyoto, Japan). IR absorption spectrum was carried out with a PerkinElmer Spectrum Two FT-IR spectrometer (PerkinElmer, Waltham, MA, USA). NMR spectra were recorded using an Agilent 600/54 Premium Compact NMR spectrometer (Agilent, Santa Clara, CA, USA) (^1^H at 600 MHz, ^13^C at 150 MHz) or a Bruker Ascend^TM^ 400 NMR spectrometer (Bruker, Germany) (^1^H at 400 MHz, ^13^C at 100 MHz) with CD_3_OD (compound **3**: CD_3_OD + DMSO-d_6_) as the solvent at 25 °C. Chemical shifts are reported in *δ* (ppm) with tetramethylsilane (TMS) as the internal standard, while coupling constants (*J*) are expressed in Hz. High-resolution electrospray ionization mass spectroscopy (HRESIMS) was measured on a Waters Q-TOF Premier mass spectrometer (Waters, Milford, MA, USA).

Column chromatography (CC) was carried out on silica gel (SiO_2_: 200–300 mesh, Qingdao Ocean Chemical Industry Co., Pingdu, Qingdao, China), polyamide (60–90 mesh, Jiangsu Changfeng Chemical Industry Co., Gulou, Nanjing, China), and MCI-gel CHP-20P (75–150 μm, Mitsubishi Chemical Co., Tokyo, Japan). Preparative HPLC was carried out on a GL3000-300 mL system instrument (Chengdu Gelai Precision Instruments Co., Ltd., Dayi, Chengdu, China) with a UV-3292 detector (detection wavelength 215 nm) and a GL C-18 column (particle size 5 μm, 50 × 450 mm), eluting with MeOH-H_2_O at 30 mL/min. TLC was performed on precoated HPTLC Fertigplatten Kieselgel 60 F_254_ plates (Merck, Rahway, NJ, USA), and the spots were visualized by spraying with 10% sulfuric acid ethanolic solution or *α*-naphthol-sulfuric acid solution and baking at 105 °C for 2–5 min. UV-vis absorbance was determined on a Spark 10M microplate reader (Tecan Trading Co. Ltd., Shanghai, China) or a UV2700 spectrophotometer (Shimadzu, Kyoto, Japan). Acetyl-coenzyme A (Ac-CoA) and NADPH were purchased from Zeye Biochemical Co., Ltd. (Shanghai, China). Methylmalonyl coenzyme A tetralithium salt hydrate (Mal-CoA) was obtained from Sigma-Aldrich (St. Louis, MO, USA). 2,2-Diphenyl-1-picrylhydrazyl (DPPH) was purchased from Macklin Biochemical Co., Ltd. (Shanghai, China). 2,2′-Azino-bis(3-ethylbenzthiazoline-6-sulphonic acid) ammonium salt (ABTS) was obtained from Aladdin Industrial Co., Ltd. (Shanghai, China).

### 2.2. Plant Material

The leaves of *L. robustum* were harvested in April 2017 from Yibin City, Sichuan Province, China, and authenticated by Professor Guo-Min Liu (Kudingcha Research Institute, Hainan University, China). A voucher specimen (No. 201704lsh) was conserved at West China School of Pharmacy, Sichuan University, China.

### 2.3. Extraction and Isolation

The fresh leaves of *L. robustum* were agitated and baked at 120 °C for 50 min and then smashed. The raw powder (7.0 kg) was extracted with 70% ethanol (28 L × 1) under reflux in a multi-function extractor for 2 h [13]. The ethanol extract was percolated and condensed in vacuo to gain a paste (2.2 kg). The paste was dissolved in 3 L 95% ethanol, and then 3 L purified water was infunded to sediment the chlorophyll. After percolation, the filtrate was condensed in vacuo to obtain a residue (1.0 kg). The residue was separated on a silica gel column, eluting with CH_2_Cl_2_-MeOH (10:0–0:10), to yield Fr. I (84 g), Fr. II (145 g), Fr. III (93 g), and Fr. IV (70 g). Fr. II was isolated repeatedly by CC on silica gel, eluting with CH_2_Cl_2_-MeOH-H_2_O (200:10:1–80:20:2) or EtOAc-MeOH-H_2_O (100:4:2–100:6:2), and then separated on polyamide column (EtOH-H_2_O, 1:9–6:4) and MCI column (MeOH-H_2_O, 3:7–8:2), and purified finally by preparative HPLC (MeOH-H_2_O, 40:60–65:35) and silica gel column (EtOAc-MeOH-H_2_O, 100:4:2–100:6:2), or recrystallized in 70% methanol, to afford **1** (107.4 mg), **4** (11.2 mg), **5** (3.5 mg), **6** (8.3 mg), **7** (14.4 mg), **8** (15.8 mg), **10** (21.3 mg), and **11** (139.4 mg). Fr. III was separated twice by CC on silica gel (EtOAc-MeOH-H_2_O, 100:4:2–100:20:10) and then subjected to polyamide column (EtOH-H_2_O, 0:10–6:4) and MCI column (MeOH-H_2_O, 3:7–5:5), and purified at last by preparative HPLC (MeOH-H_2_O, 30:70–50:50) and silica gel column (EtOAc-MeOH-H_2_O, 100:10:5), to give **2** (37.3 mg), **3** (22.4 mg), and **9** (13.5 mg).

Compound **1**: white amorphous powder. [*α*]^20^_D_ −43.9 (c 0.28, MeOH); UV (MeOH) λ_max_: (log ε) 212 (4.1), 227 (4.2), 317 (4.4) nm; IR (film) ν_max_: 3368, 2930, 1690, 1604, 1515, 1445, 1261, 1039, 981, 829 cm^−1^; ^1^H NMR (CD_3_OD, 400 MHz) data, see Table 1; ^13^C NMR (CD_3_OD, 100 MHz) data, see Table 2; HRESIMS *m*/*z* 761.2634 [M + Na]^+^ (calculated for C_35_H_46_NaO_17_, 761.2633).

Compound **2**: white amorphous powder. [*α*]^23^_D_ −62.1 (*c* 0.49, MeOH); UV (MeOH) λ_max_ (log ε): 213 (4.1), 226 (4.2), 318 (4.4) nm; IR (film) ν_max_: 3356, 2931, 1693, 1630, 1603, 1515, 1448, 1263, 1040, 982, 834, 803 cm^−^^1^; ^1^H NMR (CD_3_OD, 400 MHz) data, see Table 1; ^13^C NMR (CD_3_OD, 150 MHz) data, see Table 2; HRESIMS *m*/*z* 793.2536 [M + Na]^+^ (calculated for C_35_H_46_NaO_19_, 793.2531).

Compound **3**: yellow amorphous powder. [*α*]^2^^3^_D_ −46.0 (*c* 0.45, MeOH); UV (MeOH) λ_max_ (log ε): 213 (4.1), 227 (4.2), 316 (4.4) nm; IR (film) ν_max_: 3402, 1652, 1604, 1048, 1029, 823, 761 cm^−^^1^; ^1^H NMR (CD_3_OD + DMSO-d_6_, 400 MHz) data, see Table 1; ^13^C NMR (CD_3_OD + DMSO-d_6_, 100 MHz) data, see Table 2; HRESIMS *m*/*z* 791.2371 [M + Na]^+^ (calculated for C_35_H_44_NaO_19_, 791.2374).

Compound **4**: white amorphous powder. [*α*]^2^^0^_D_ −122.4 (*c* 0.25, MeOH); UV (MeOH) λ_max_ (log ε): 210 (3.9), 230 (3.9), 315 (4.4) nm; IR (film) ν_max_: 3360, 2929, 1695, 1603, 1449, 1330, 1259, 1157, 1021, 912, 833, 741, 699 cm^−^^1^; ^1^H NMR (CD_3_OD, 400 MHz) data, see Table 3; ^13^C NMR (CD_3_OD, 100 MHz) data, see Table 4; HRESIMS *m/z* 585.1943 [M + Na]^+^ (calculated for C_28_H_34_ NaO_12_, 585.1948).

Compound **5**: yellowish amorphous powder. [*α*]^2^^0^_D_ −18.5 (*c* 0.18, MeOH); UV (MeOH) λ_max_ (log ε): 210 (3.9), 230 (3.9), 315 (4.4) nm; IR (film) ν_max_: 3369, 2925, 2854, 1706, 1605, 1512, 1452, 1164, 1038, 836, 700 cm^−^^1^; ^1^H NMR (CD_3_OD, 400 MHz) data, see Table 3; ^13^C NMR (CD_3_OD, 150 MHz) data, see Table 4; HRESIMS *m*/*z* 585.1947 [M + Na]^+^ (calculated for C_28_H_34_NaO_12_, 585.1948).

Compound **6**: white amorphous powder. [*α*]^2^^0^_D_ −18.5 (*c* 0.18, MeOH); UV (MeOH) λ_max_ (log ε): 210 (3.9), 230 (3.9), 316 (4.4) nm; IR (film) ν_max_: 3369, 2925, 2854, 1706, 1605, 1512, 1452, 1164, 1038, 836, 700 cm^−^^1^; ^1^H NMR (CD_3_OD, 600 MHz) data, see Table 3; ^13^C NMR (CD_3_OD, 100 MHz) data, see Table 4; HRESIMS *m*/*z* 585.1949 [M + Na]^+^ (calculated for C_28_H_34_NaO_12_, 585.1948).

### 2.4. Acid Hydrolysis of Compounds ***1**–**6***

Compounds **1**–**6** (2 mg), dissolved in 0.1 mL MeOH, were injected into 2 mL H_2_SO_4_ aqueous solution (1 M) and hydrolyzed at 95 °C for 6 h, respectively. Then, 2 mL Ba(OH)_2_ solution (1 M) was added. The hydrolyzed solution was filtered and condensed. The monosaccharides in the condensed solution were affirmed by TLC (EtOAc- MeOH-HOAc-H_2_O, 8:1:1:0.7, 2 developments) with authentic samples [13]. The *R_f_* values of D-mannose, D-glucose, and L-rhamnose were 0.46, 0.43, and 0.73, respectively.

### 2.5. Enzymatic Hydrolysis of Compounds ***2***

Compound **2** (20 mg) and cellulase (30 mg) were added to 12 mL HOAc-NaOAc buffer solution (pH 5.0) and kept at 37 °C for 6 h. The hydrolyzed product was extracted with EtOAc and purified on a silica gel column (eluting with EtOAc) to afford (*R*)-(-)-l-(3,4-dihydroxyphenyl)ethane-l,2-diol and (*S*)-(+)-l-(3,4-dihydroxyphenyl)ethane- l,2-diol (9:11) confirmed by [*α*]^27^_D_ +4.8 (*c* 0.15, EtOAc) [17].

### 2.6. Determination of Bioactivities

The inhibitory effects on FAS, *α*-glucosidase and *α*-amylase, and the DPPH and ABTS radical scavenging activities of compounds **1**–**11** were determined by the reported methods [12,13,18,19], while orlistat, acarbose, and L-(+)-ascorbic acid were applied as the positive controls, respectively (Supplementary Material S1).

### 2.7. Statistical Analyses

Statistical analyses were performed on GraphPad Prism 5.01. All samples were determined in triplicate. The IC_50_ (the ultimate concentration of sample needed to inhibit 50% of enzyme activity or clear away 50% of free radicals) was acquired by plotting the inhibition or scavenging percentage of every sample against its concentration. The results are recorded as mean ± standard deviation (SD). Differences of means between several groups were analyzed by one-way analysis of variance (ANOVA) on the statistical package SPSS 25.0. The differences between groups were deemed to be significant when *p* < 0.05.

## 3. Results and Discussion

### 3.1. Identification of Compounds ***1**–**11***

**Figure 2:**
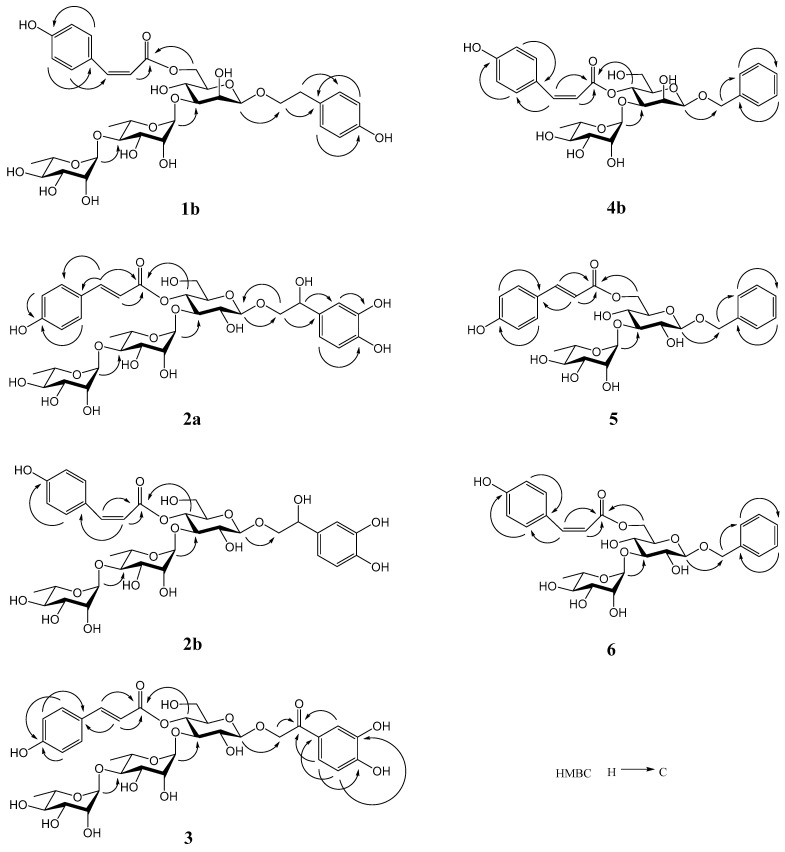
Key HMBC correlations of compounds **1**–**6**.

Compound **1** was analyzed as C_3__5_H_46_O_1__7_ by HRESIMS (*m*/*z* 761.2634 [M + Na]^+^, calculated 761.2633 for C_3__5_H_46_NaO_1__7_). The NMR spectra of **1** showed 2 stereoisomers **1a** and **1b** (5:1). The ^1^H and ^13^C NMR data of **1a** (Supplementary Material S2.) was in agreement with those of 2-(4-hydroxyphenyl)ethyl 3-*O*-[*α*-l-rhamnopyranosyl-(1→4)-*α*-l-rhamnopyranosyl]-6-*O*-(*trans*-*p*-coumaroyl)-*O*-*β*-D-mannopyranoside (ligurobustoside R) [12]. The NMR data of **1b** (Table 1 and Table 2) were similar to those of **1a**, except the *trans*-*p*-coumaroyl [*δ*_H_ 7.62, 6.35 (1H each, d, *J* = 16.0 Hz, H-7’’’’, H-8’’’’)] in **1a** was replaced by the *cis*-*p*-coumaroyl [*δ*_H_ 6.86, 5.79 (1H each, d, *J* = 12.8 Hz, H-7’’’’, H-8’’’’)] in **1b**. The acid hydrolysis experiment of **1** gave D-mannose, and L-rhamnose was affirmed by TLC. The HMBC experiment of **1b** (Figure 2) displayed the long-distance correlations: between *δ*_H_ 4.28 (H-1’ of mannosyl) and *δ*_C_ 72.3 (C-8 of aglycone), between *δ*_H_ 5.17 (H-1’’ of inner rhamnosyl) and *δ*_C_ 83.6 (C-3’ of mannosyl), between *δ*_H_ 5.19 (H-1’’’ of outer rhamnosyl) and *δ*_C_ 81.1 (C-4’’ of inner rhamnosyl), and between *δ*_H_ 4.29 (H-6’a of mannosyl), 4.46 (H-6’b of mannosyl) and *δ*_C_ 168.1 (carbonyl of coumaroyl). The ^1^H and ^13^C NMR signals of **1b** were assigned by the HMBC experiment (Figure S1). So **1b** was identified as 2-(4-hydroxyphenyl)ethyl 3-*O*-[*α*-L-rhamnopyranosyl-(1→4)-*α*-L-rhamnopyranosyl]-6-*O*-(*cis*-*p*-coumaroyl)-*O*-*β*-d-mannopyranoside. It is a novel phenylethanoid glycoside named ligurobustoside R_1_. In conclusion, compound **1** is a mixture of ligurobustosides R and R_1_.

Compound **2** was analyzed as C_3__5_H_4__6_O_1__9_ by HRESIMS (*m*/*z* 793.2536 [M + Na]^+^, calculated 793.2531 for C_3__5_H_4__6_NaO_1__9_). The NMR spectra of **2** showed 2 stereoisomers **2a** and **2b** (10:3). The ^1^H NMR spectrum of **2a** (Table 1) revealed the following signals: (1) a 4-substituted phenyl at *δ*_H_ 6.82, 7.49 (2H each, d, *J* = 8.8 Hz); (2) a 3,4-disubstituted phenyl at *δ*_H_ 6.72 (1H, d, *J* = 2.0 Hz), 6.74 (1H, d, *J* = 8.0 Hz), and 6.83 (1H, dd, *J* = 8.0, 2.0 Hz); (3) a trans double bond at *δ*_H_ 7.67 and 6.33 (1H each, d, *J* = 16.0 Hz); (4) three anomeric protons at *δ*_H_ 4.41 (1H, d, *J* = 8.0 Hz), 5.04 (1H, d, *J* = 2.0 Hz), and 5.22 (1H, d, *J* = 2.0 Hz); (5) a methylene at *δ*_H_ 3.56–3.72 (1H, m) and 3.90–3.98 (1H, m), a methyne at *δ*_H_ 4.75 (1H, dd, *J* = 9.6, 3.2 Hz), and two methyl groups at *δ*_H_ 1.04 (3H, d, *J* = 6.0 Hz) and 1.09 (3H, d, *J* = 6.0 Hz). The ^13^C NMR spectrum of **2a** (Table 2) showed a carbonyl at *δ*_C_ 168.1, 2 phenyl groups at *δ*_C_ 114.6–161.5, a double bond at *δ*_C_ 114.7 and 147.6, 3 anomeric carbons at *δ*_C_ 102.6–104.6, 13 sugar carbons at *δ*_C_ 62.2–81.6, a methylene at *δ*_C_ 76.7, a methyne at *δ*_C_ 74.2, and 2 methyl groups at *δ*_C_ 17.7 and 19.1. The above ^1^H and ^13^C NMR features of **2a** were related closely to those of (2*R*)-2-hydroxy-2-(3,4-dihydroxyphenyl)ethyl 3-*O*-[*α*-L-rhamnopyranosyl-(1→4)-*α*-l-rhamnopyranosyl]-4-*O*-(*trans*-caffeoyl)-*O*-*β*-d-glucopyranoside (ligurobustoside P) [7], except that the *trans*-caffeoyl in ligurobustoside P was replaced by the *trans*-*p*-coumaroyl in **2a**. The acid hydrolysis experiment of **2** gave d-glucose, and l-rhamnose was affirmed by TLC. Furthermore, the HMBC experiment of **2a** (Figure 2) displayed the long-distance correlations: between *δ*_H_ 4.41 (H-1’ of glucosyl) and *δ*_C_ 76.7 (C-8 of aglycone), between *δ*_H_ 5.22 (H-1’’ of inner rhamnosyl) and *δ*_C_ 81.2 (C-3’ of glucosyl), between *δ*_H_ 5.04 (H-1’’’ of outer rhamnosyl) and *δ*_C_ 81.6 (C-4’’ of inner rhamnosyl), and between *δ*_H_ 4.95 (H-4’ of glucosyl) and *δ*_C_ 168.1 (carbonyl of coumaroyl). The ^1^H and ^13^C NMR signals of **2** were assigned by ^1^H-^1^H COSY, HSQC, and HMBC experiments (Figure S2). Thus, the plane structure of **2a** was elucidated as 2-hydroxy-2-(3,4-dihydroxyphenyl)ethyl 3-*O*-[*α*-L-rhamnopyranosyl-(1→4)-*α*-L-rhamnopyranosyl]-4-*O*-(*trans*-*p*-coumaroyl)-*O*-*β*-d-glucopyranoside.

The NMR data of **2b** (Table 1 and Table 2) were similar to those of **2a**, except the *trans*-*p*-coumaroyl in **2a** was replaced by the *cis*-*p*-coumaroyl [*δ*_H_ 6.99, 5.76 (1H each, d, *J* = 12.8 Hz, H-7’’’’, H-8’’’’)] in **2b**. The HMBC experiment of **2b** (Figure 2) displayed the long-distance correlations: between *δ*_H_ 4.43 (H-1’ of glucosyl) and *δ*_C_ 76.7 (C-8 of aglycone), between *δ*_H_ 5.21 (H-1’’ of inner rhamnosyl) and *δ*_C_ 81.1 (C-3’ of glucosyl), between *δ*_H_ 5.06 (H-1’’’ of outer rhamnosyl) and *δ*_C_ 81.5 (C-4’’ of inner rhamnosyl), and between *δ*_H_ 4.90 (H-4’ of glucosyl) and *δ*_C_ 166.8 (carbonyl of coumaroyl). Therefore, the plane structure of **2b** was identified as 2-hydroxy-2-(3,4-dihydroxyphenyl)ethyl 3-*O*-[*α*-L-rhamnopyranosyl-(1→4)-*α*-L-rhamnopyranosyl]-4-*O*-(*cis*-*p*-coumaroyl)-*O*-*β*-d-glucopyranoside.

In addition, the enzymatic hydrolysis experiment of **2** gave (*R*)-(-)-l-(3,4-dihydroxyphenyl)ethane-l,2-diol and (*S*)-(+)-l-(3,4-dihydroxyphenyl)ethane-l,2-diol (9:11), meaning that *R*/*S* (9:11) was not equal to **2a**/**2b** (10:3). Based on the above evidence, compound **2** was characterized as a mixture of (2*R*/*S*)-2-hydroxy-2-(3,4-dihydroxyphenyl)ethyl 3-*O*-[*α*-L-rhamnopyranosyl-(1→4)-*α*-L-rhamnopyranosyl]-4-*O*-(*trans*-*p*-coumaroyl)-*O*-*β*-d-glucopyranoside and (2*R*/*S*)-2-hydroxy-2-(3,4-dihydroxyphenyl)ethyl 3-*O*-[*α*-L-rhamnopyranosyl-(1→4)-*α*-L-rhamnopyranosyl]-4-*O*-(*cis*-*p*-coumaroyl)-*O*-*β*-d-glucopyranoside. It is a novel phenylethanoid glycoside named ligurobustoside R_2–3_.

Compound **3** was determined as C_3__5_H_4__4_O_1__9_ by HRESIMS (*m*/*z* 791.2371 [M + Na]^+^, calculated 791.2374 for C_3__5_H_4__4_NaO_1__9_). The ^1^H NMR spectrum of **3** (Table 1) showed the following signals: (1) a 4-substituted phenyl at *δ*_H_ 6.87, 7.54 (2H each, d, *J* = 8.4 Hz); (2) a 3,4-disubstituted phenyl at *δ*_H_ 6.89 (1H, d, *J* = 8.4 Hz), 7.46 (1H, br. s) and 7.47 (1H, br. d, *J* = 8.4 Hz); (3) a trans double bond at *δ*_H_ 7.68 and 6.37 (1H each, d, *J* = 16.0 Hz); (4) three anomeric protons at *δ*_H_ 4.54 (1H, d, *J* = 7.6 Hz), 5.06 (1H, br. s) and 5.22 (1H, br. s); (5) a methylene at *δ*_H_ 4.98 and 5.26 (1H each, d, *J* = 16.8 Hz), and two methyl groups at *δ*_H_ 1.06 (3H, d, *J* = 6.0 Hz) and 1.10 (3H, d, *J* = 6.0 Hz). The ^13^C NMR spectrum of **3** (Table 2) revealed 2 carbonyl groups at *δ*_C_ 167.6 and 196.4, 2 phenyl groups at *δ*_C_ 115.8–161.4, a double bond at *δ*_C_ 114.9 and 147.3, 3 anomeric carbons at *δ*_C_ 102.6–103.9, 13 sugar carbons at *δ*_C_ 62.2–81.2, a methylene at *δ*_C_ 72.2, and 2 methyl groups at *δ*_C_ 18.1 and 19.4. The above ^1^H and ^13^C NMR characteristics of **3** were similar to those of **2a**, except that the methyne (C-7 of aglycone) linking with hydroxy in **2a** was replaced by the carbonyl in **3**. The acid hydrolysis experiment of **3** afforded D-glucose and L-rhamnose affirmed by TLC. Additionally, the HMBC experiment of **3** (Figure 2) displayed the long-distance correlations: between *δ*_H_ 7.46 (H-2), 7.47 (H-6), 4.98 (H-8a), 5.26 (H-8b) and *δ*_C_ 196.4 (C-7), between *δ*_H_ 4.54 (H-1’ of glucosyl) and *δ*_C_ 72.2 (C-8 of aglycone), between *δ*_H_ 5.22 (H-1’’ of inner rhamnosyl) and *δ*_C_ 81.1 (C-3’ of glucosyl), between *δ*_H_ 5.06 (H-1’’’ of outer rhamnosyl) and *δ*_C_ 81.2 (C-4’’ of inner rhamnosyl), and between *δ*_H_ 4.96 (H-4’ of glucosyl) and *δ*_C_ 167.6 (carbonyl of coumaroyl). The ^1^H and ^13^C NMR signals of **3** were assigned by ^1^H-^1^H COSY, HSQC, and HMBC experiments (Figure S3). Therefore, compound **3** was determined to be 2-(3,4-dihydroxyphenyl)-2-oxoethyl 3-*O*-[*α*-l-rhamnopyranosyl-(1→4)-*α*-L-rhamnopyranosyl]-4-*O*-(*trans*-*p*-coumaroyl)-*O*-*β*-d-glucopyranoside. It is a novel phenylethanoid glycoside named ligurobustoside R_4_.

Compound **4** was determined as C_28_H_34_O_1__2_ by HRESIMS (*m*/*z* 585.1943 [M + Na]^+^, calculated 585.1948 for C_28_H_34_NaO_1__2_). The NMR spectra of **4** exhibited 2 stereoisomers **4a** and **4b** (2:1). The ^1^H and ^13^C NMR data of **4a** (Supplementary Material S2.) was in accordance with those of benzyl 3-*O*-(*α*-L-rhamnopyranosyl)-4-*O*-(*trans*-*p*-coumaroyl)-*O*-*β*-d-mannopyranoside (ligurobustoside S) [12]. The NMR data of **4b** (Table 3 and Table 4) were very similar to those of **4a**, except the *trans*-*p*-coumaroyl [*δ*_H_ 7.67, 6.35 (1H each, d, *J* = 16.0 Hz, H-7’’’, H-8’’’)] in **4a** was replaced by the *cis*-*p*-coumaroyl [*δ*_H_ 6.95, 5.80 (1H each, d, *J* = 12.8 Hz, H-7’’’, H-8’’’)] in **4b**. The acid hydrolysis experiment of **4** offered D-mannose and L-rhamnose confirmed by TLC. The HMBC experiment of **4b** (Figure 2) displayed the long-distance correlations: between *δ*_H_ 4.42 (H-1’ of mannosyl) and *δ*_C_ 72.0 (C-7 of aglycone), between *δ*_H_ 5.16 (H-1’’ of rhamnosyl) and *δ*_C_ 81.6 (C-3’ of mannosyl), and between *δ*_H_ 4.90 (H-4’ of mannosyl) and *δ*_C_ 166.9 (carbonyl of coumaroyl). The ^1^H and ^13^C NMR signals of **4** were assigned by the HMBC experiment (Figure S4). So **4b** was identified as benzyl 3-*O*-(*α*-L-rhamnopyranosyl)-4-*O*-(*cis*-*p*-coumaroyl)-*O*-*β*-d-mannopyranoside. It is a new phenylmethanoid glycoside named ligurobustoside S_1_. In sum, compound **4** is a mixture of ligurobustosides S and S_1_.

Compound **5** was determined as C_28_H_34_O_1__2_ by HRESIMS (*m*/*z* 585.1947 [M + Na]^+^, calculated 585.1948 for C_28_H_34_NaO_1__2_). The ^1^H NMR spectrum of **5** (Table 3) showed the following signals: (1) a 4-substituted phenyl at *δ*_H_ 6.79, 7.46 (2H each, d, *J* = 8.4 Hz); (2) a phenyl at *δ*_H_ 7.26 (1H, br. d, *J* = 7.2 Hz), 7.30 (2H, br. t, *J* = 7.2 Hz), and 7.39 (2H, br. d, *J* = 7.2 Hz); (3) a trans double bond at *δ*_H_ 7.66 and 6.38 (1H each, d, *J* = 16.0 Hz); (4) two anomeric protons at *δ*_H_ 4.38 (1H, d, *J* = 8.0 Hz) and 5.17 (1H, d, *J* = 2.0 Hz); (5) a methylene at *δ*_H_ 4.65 and 4.87 (1H each, d, *J* = 12.0 Hz), and a methyl at *δ*_H_ 1.24 (3H, d, *J* = 6.4 Hz). The ^13^C NMR spectrum of **5** (Table 4) revealed a carbonyl at *δ*_C_ 169.2, two phenyl groups at *δ*_C_ 117.1–162.2, a double bond at *δ*_C_ 114.5 and 147.0, two anomeric carbons at *δ*_C_ 102.7 and 103.1, nine sugar carbons at *δ*_C_ 64.6–83.9, a methylene at *δ*_C_ 72.0, and a methyl at *δ*_C_ 17.9. The above ^1^H and ^13^C NMR characteristics of **5** were similar to those of benzyl 6-*O*-[(*E*)-3-(3,4-dihydroxyphenyl)-prop-2-enoyl]-3-*O*-(*α*-L-rhamnopyranosyl)-*O*-*β*-d-glucopyranoside (salsaside A) [20], except the *trans*-caffeoyl in salsaside A was replaced by the *trans*-*p*-coumaroyl in **5**. The acid hydrolysis experiment of **5** yielded d-glucose and L-rhamnose identified by TLC. The HMBC experiment of **5** (Figure 2) displayed the long-distance correlations: between *δ*_H_ 4.38 (H-1’ of glucosyl) and *δ*_C_ 72.0 (C-7 of aglycone), between *δ*_H_ 5.17 (H-1’’ of rhamnosyl) and *δ*_C_ 83.9 (C-3’ of glucosyl), and between *δ*_H_ 4.38 (H-6’a of glucosyl), 4.52 (H-6’b of glucosyl) and *δ*_C_ 169.2 (carbonyl of coumaroyl). The ^1^H and ^13^C NMR signals of **5** were assigned by ^1^H-^1^H COSY, HSQC, and HMBC experiments (Figure S5). Therefore, compound **5** was elucidated to be benzyl 3-*O*-(*α*-L-rhamnopyranosyl)-6-*O*-(*trans*-*p*-coumaroyl)-*O*-*β*-d-glucopyranoside. It is a new phenylmethanoid glycoside named ligurobustoside S_2_.

Compound **6** was analyzed as C_28_H_34_O_1__2_ by HRESIMS (*m*/*z* 585.1949 [M + Na]^+^, calculated 585.1948 for C_28_H_34_NaO_1__2_). The ^1^H and ^13^C NMR data of **6** (Table 3 and Table 4) were related closely to those of **5**, except the *trans*-*p*-coumaroyl [*δ*_H_ 7.66, 6.38 (1H each, d, *J* = 16.0 Hz, H-7’’’, H-8’’’)] in **5** was replaced by the *cis*-*p*-coumaroyl [*δ*_H_ 6.90, 5.82 (1H each, d, *J* = 13.2 Hz, H-7’’’, H-8’’’)] in **6**. The acid hydrolysis experiment of **6** yielded D-glucose and L-rhamnose affirmed by TLC. The HMBC experiment of **6** (Figure 2) showed the long-distance correlations: between *δ*_H_ 4.33 (H-1’ of glucosyl) and *δ*_C_ 72.0 (C-7 of aglycone), between *δ*_H_ 5.15 (H-1’’ of rhamnosyl) and *δ*_C_ 84.1 (C-3’ of glucosyl), and between *δ*_H_ 4.30 (H-6’a of glucosyl), 4.50 (H-6’b of glucosyl) and *δ*_C_ 168.2 (carbonyl of coumaroyl). The ^1^H and ^13^C NMR signals of **6** were assigned by ^1^H-^1^H COSY, HSQC, and HMBC experiments (Figure S6). Thus, compound **6** was identified as benzyl 3-*O*-(*α*-L-rhamnopyranosyl)-6-*O*-(*cis*-*p*-coumaroyl)-*O*-*β*-d-glucopyranoside. It is a new phenylmethanoid glycoside named ligurobustoside S_3_.

Compounds **7–11** (^1^H, ^13^C NMR data see S2.) were identified as reported ligupurpuroside B (**7**) [21], *cis*-ligupurpuroside B (**8**) [21,22], ligurobustoside N (**9**) [1], osmanthuside D (**10**) [23], and (*Z*)-osmanthuside B_6_ (**11**) [23,24], by comparison with published NMR data and 2D-NMR experiments (^1^H-^1^H COSY, HSQC, and HMBC). Compounds **8**, **10,** and **11** were isolated from this plant for the first time.

### 3.2. The Bioactivities of Compounds ***1**–**11***

Compounds **1**–**11** from the leaves of *L. robustum* were measured for the inhibitory effects on FAS, *α*-glucosidase, *α*-amylase, and antioxidant activities. The results of the bioactivity assays are shown in Table 5. As shown in Table 5, the FAS inhibitory effect of compound **11** (IC_50_: 4.55 ± 0.35 μM) was as strong as the positive control orlistat (IC_50_: 4.46 ± 0.13 μM), while the FAS inhibitory effects of compounds **4** (IC_50_: 6.49 ± 0.27 μM) and **9** (IC_50_: 5.61 ± 0.44 μM) were weaker than orlistat; the *α*-glucosidase inhibitory effects of compounds **3** and **5** were moderate and weaker than the positive control acarbose; the *α*-amylase inhibitory effects of compounds **10** and **11** were moderate and weaker than the positive control acarbose; the DPPH radical scavenging activities of compounds **2, 3,** and **9** (IC_50_: 23.83 ± 0.89~43.17 ± 1.06 μM) were weaker than the positive control L-(+)-ascorbic acid (IC_50_: 13.66 ± 0.13 μM); the ABTS radical scavenging activities of compounds **3**, **5**, and **7**–**11** (IC_50_: 2.68 ± 0.05~4.86 ± 0.06 μM) were stronger than the positive control L-(+)-ascorbic acid (IC_50_: 10.06 ± 0.19 μM).

The previous study revealed that FAS was a potential therapeutic target for anti-obesity drugs [13,18]; *α*-glucosidase and *α*-amylase were two important targets to prevent diabetes and obesity [12,25]; and reactive oxygen species played an important role in the initiation and progression of diabetes [12,26]. Consequently, antioxidants **3**–**5**, **9**, and **10**, with some FAS, *α*-glucosidase, and *α*-amylase inhibitory activities [12], might be a part of the effective ingredients for *L. robustum* to prevent diabetes and obesity.

## 4. Conclusions

In summary, the phytochemical investigation on the leaves of *L. robustum* resulted in the isolation of eight phenylethanoid glycosides (**1**–**3**, **7**–**11**) and three phenylmethanoid glycosides (**4**–**6**), including six novel compounds (**1b**,**2**,**3**,**4b**,**5**,**6**) identified with spectroscopic method (^1^H, ^13^C NMR, ^1^H-^1^H COSY, HSQC, HMBC, HRESIMS), and physical and chemical methods. The biological assays showed that the FAS inhibitory effect of compound **11** (IC_50_: 4.55 ± 0.35 μM) was as strong as the positive control orlistat (IC_50_: 4.46 ± 0.13 μM); the *α*-glucosidase inhibitory effects of compounds **3** and **5**, and the *α*-amylase inhibitory effects of compounds **10** and **11** were moderate; the DPPH radical scavenging activities of compounds **2**, **3,** and **9** (IC_50_: 23.83 ± 0.89~43.17 ± 1.06 μM) were weaker than the positive control l-(+)-ascorbic acid (IC_50_: 13.66 ± 0.13 μM); the ABTS radical scavenging activities of compounds **3**, **5**, and **7**–**11** (IC_50_: 2.68 ± 0.05~4.86 ± 0.06 μM) were stronger than the positive control l-(+)-ascorbic acid (IC_50_: 10.06 ± 0.19 μM). Together this work and previous studies [12,13], phenylethanoid, phenylmethanoid, and monoterpenoid glycosides were believed as the main anti-obesity and anti-diabetes components of *L. robustum*. This research offered a theoretical basis for the leaves of *L. robustum* as a functional tea to prevent obesity and diabetes.

## Figures and Tables

**Table 1:** ^1^H NMR (400 MHz) data of compounds **1**–**3** from *L. robustum ^a^*.

No	1b *^b^*	2a *^b^*	2b *^b^*	3 *^c^*
2	7.01 d (8.0)	6.72 d (2.0)	6.72 d (2.0)	7.46 br. s
3	6.67 d (8.0)			
5	6.67 d (8.0)	6.74 d (8.0)	6.74 d (8.0)	6.89 d (8.4)
6	7.01 d (8.0)	6.83 dd (8.0, 2.0)	6.83 dd (8.0, 2.0)	7.47 br. d (8.4)
7	2.83 t (7.2)	4.75 dd (9.6, 3.2)	4.75 dd (9.6, 3.2)	
8	3.72 m	3.56–3.72 m	3.56–3.72 m	4.98 d (16.8)
	3.96 m	3.90–3.98 m	3.90–3.98 m	5.26 d (16.8)
Glc or Man				
1’	4.28 d (7.6)	4.41 d (8.0)	4.43 d (8.0)	4.54 d (7.6)
2’	3.31 m	3.46 m	3.45 m	3.52 m
3’	3.54 m	3.83 m	3.80 m	3.87 m
4’	3.38 m	4.95 t (9.6)	4.90 t (9.6)	4.96 t (9.6)
5’	3.53 m	3.56 m	3.51 m	3.61 m
6’	4.29 dd (11.6, 6.4)	3.53 m	3.53 m	3.54 m
	4.46 dd (11.6, 2.0)	3.61 m	3.61 m	3.61 m
Inner-Rha				
1’’	5.17 d (2.0)	5.22 d (2.0)	5.21 d (2.0)	5.22 br. s
2’’	3.89 m	3.88 dd (3.2, 2.0)	3.82 dd (3.2, 2.0)	3.87 m
3’’	3.84 dd (9.6, 3.2)	3.68 dd (9.2, 3.2)	3.68 dd (9.2, 3.2)	3.66 m
4’’	3.53 m	3.40 m	3.40 m	3.40 m
5’’	4.10 m	3.60 m	3.60 m	3.60 m
6’’	1.28 d (6.0)	1.09 d (6.0)	1.08 d (6.0)	1.10 d (6.0)
Outer-Rha				
1’’’	5.19 d (1.6)	5.04 d (2.0)	5.06 d (2.0)	5.06 br. s
2’’’	3.94 m	3.90 dd (3.2, 2.0)	3.90 dd (3.2, 2.0)	3.88 m
3’’’	3.60 dd (9.6, 3.2)	3.51 m	3.51 m	3.49 m
4’’’	3.39 m	3.32 m	3.32 m	3.32 m
5’’’	3.70 m	3.46 m	3.46 m	3.46 m
6’’’	1.25 d (6.4)	1.04 d (6.0)	1.04 d (6.0)	1.06 d (6.0)
Cou				
2’’’’	7.62 d (8.4)	7.49 d (8.8)	7.72 d (8.8)	7.54 d (8.4)
3’’’’	6.75 d (8.4)	6.82 d (8.8)	6.77 d (8.8)	6.87 d (8.4)
5’’’’	6.75 d (8.4)	6.82 d (8.8)	6.77 d (8.8)	6.87 d (8.4)
6’’’’	7.62 d (8.4)	7.49 d (8.8)	7.72 d (8.8)	7.54 d (8.4)
7’’’’	6.86 d (12.8)	7.67 d (16.0)	6.99 d (12.8)	7.68 d (16.0)
8’’’’	5.79 d (12.8)	6.33 d (16.0)	5.76 d (12.8)	6.37 d (16.0)

*^a^* Coupling constants (*J* values in Hz) are shown in parentheses. *^b^* In CD_3_OD. *^c^* In CD_3_OD + DMSO-d_6_.

**Table 2:** ^13^C NMR data of compounds **1–3** from *L. robustum*.

No	1b *^a^*	2a *^b^*	2b *^b^*	3 *^c^*
1	130.6	133.6	133.6	127.9
2	130.9	119.0	119.0	115.8
3	116.1	146.3	146.3	146.7
4	156.7	146.1	146.1	152.9
5	116.1	116.2	116.2	117.0
6	130.9	114.6	114.6	122.9
7	36.4	74.2	74.2	196.4
8	72.3	76.7	76.7	72.2
Glc or Man				
1’	104.2	104.6	104.4	103.9
2’	75.7	76.4	76.4	76.2
3’	83.6	81.2	81.1	81.1
4’	70.4	70.3	70.1	70.4
5’	75.2	76.1	75.9	76.2
6’	64.4	62.2	62.3	62.2
Inner-Rha				
1’’	103.2	102.6	102.7	102.6
2’’	72.8	72.8	72.8	72.7
3’’	73.0	72.6	72.6	72.6
4’’	81.1	81.6	81.5	81.2
5’’	68.4	68.9	68.6	68.8
6’’	18.6	19.1	18.9	19.4
Outer-Rha				
1’’’	102.4	103.5	103.4	103.3
2’’’	72.3	72.3	72.2	72.3
3’’’	72.3	72.3	72.2	72.3
4’’’	73.8	73.8	73.9	73.6
5’’’	70.4	70.3	70.1	70.3
6’’’	17.8	17.7	17.8	18.1
Cou				
1’’’’	127.5	126.9	127.5	126.9
2’’’’	133.7	131.5	134.3	131.5
3’’’’	115.9	117.1	115.0	117.2
4’’’’	160.1	161.5	160.3	161.4
5’’’’	115.9	117.1	115.0	117.2
6’’’’	133.7	131.5	134.3	131.5
7’’’’	145.3	147.6	147.5	147.3
8’’’’	116.3	114.7	115.7	114.9
CO	168.1	168.1	166.8	167.6

*^a^* At 100 MHz, in CD_3_OD. *^b^* At 150 MHz, in CD_3_OD. *^c^* At 100 MHz, in CD_3_OD + DMSO-d_6_.

**Table 3:** ^1^H NMR data of compounds **4**–**6** from *L. robustum* in CD_3_OD *^a^*.

No	4b *^b^*	5 *^b^*	6 *^c^*
2	7.43 br. d (7.2)	7.39 br. d (7.2)	7.36 br. d (7.8)
3	7.35 br. t (7.2)	7.30 br. t (7.2)	7.29 br. t (7.8)
4	7.28 br. d (7.2)	7.26 br. d (7.2)	7.25 br. d (7.8)
5	7.35 br. t (7.2)	7.30 br. t (7.2)	7.29 br. t (7.8)
6	7.43 br. d (7.2)	7.39 br. d (7.2)	7.36 br. d (7.8)
7	4.68 d (11.6)	4.65 d (12.0)	4.59 d (12.0)
	4.96 d (11.6)	4.87 d (12.0)	4.80 d (12.0)
Glc or Man			
1’	4.42 d (8.0)	4.38 d (8.0)	4.33 d (7.8)
2’	3.46 dd (9.2, 8.0)	3.38 m	3.37 m
3’	3.76 t (9.2)	3.52 t (8.8)	3.49 t (9.0)
4’	4.90 m	3.43 m	3.37 m
5’	3.54 m	3.52 m	3.49 m
6’	3.56 m	4.38 dd (12.0, 3.6)	4.30 dd (12.0, 6.0)
	3.64 m	4.52 dd (12.0, 2.0)	4.50 dd (12.0, 1.8)
Rha			
1’’	5.16 d (1.6)	5.17 d (2.0)	5.15 d (1.8)
2’’	3.92 dd (3.2, 1.6)	3.94 dd (3.6, 2.0)	3.93 dd (3.0, 1.8)
3’’	3.58 m	3.70 dd (9.6, 3.6)	3.70 dd (9.6, 3.0)
4’’	3.29 t (9.6)	3.39 m	3.39 t (9.6)
5’’	3.56 m	4.00 dd (9.6, 6.4)	3.99 dd (9.6, 6.0)
6’’	1.16 d (6.0)	1.24 d (6.4)	1.24 d (6.0)
Cou			
2’’’	7.73 d (8.8)	7.46 d (8.4)	7.66 d (8.4)
3’’’	6.76 d (8.8)	6.79 d (8.4)	6.76 d (8.4)
5’’’	6.76 d (8.8)	6.79 d (8.4)	6.76 d (8.4)
6’’’	7.73 d (8.8)	7.46 d (8.4)	7.66 d (8.4)
7’’’	6.95 d (12.8)	7.66 d (16.0)	6.90 d (13.2)
8’’’	5.80 d (12.8)	6.38 d (16.0)	5.82 d (13.2)

*^a^* Coupling constants (*J* values in Hz) are shown in parentheses. *^b^* At 400 MHz. *^c^* At 600 MHz.

**Table 4:** ^13^C NMR data of compounds **4–6** from *L. robustum* in CD_3_OD.

No	4b *^a^*	5 *^b^*	6 *^a^*
1	139.0	138.8	138.8
2	129.3	129.3	129.4
3	129.1	129.2	129.3
4	128.7	128.8	128.8
5	129.1	129.2	129.3
6	129.3	129.3	129.4
7	72.0	72.0	72.0
Glc or Man			
1’	103.2	103.1	103.1
2’	76.2	75.7	75.6
3’	81.6	83.9	84.1
4’	70.6	70.4	70.5
5’	76.1	75.5	75.4
6’	62.4	64.6	64.5
Rha			
1’’	103.0	102.7	102.8
2’’	72.3	72.3	72.4
3’’	72.0	72.2	72.3
4’’	73.8	74.0	74.0
5’’	70.4	70.0	70.0
6’’	18.2	17.9	17.9
Cou			
1’’’	127.5	126.7	127.4
2’’’	134.2	131.3	133.8
3’’’	115.8	117.1	116.1
4’’’	160.4	162.2	160.6
5’’’	115.8	117.1	116.1
6’’’	134.2	131.3	133.8
7’’’	147.3	147.0	145.3
8’’’	115.8	114.5	116.1
CO	166.9	169.2	168.2

*^a^* At 100 MHz. *^b^* At 150 MHz.

**Table 5:** Results of the bioactivity assays of compounds **1**–**11** from *L. robustum ^a^*.

Compounds	FAS IC_50_ (μM) *^b^*	*α*-Glucosidase Inhibition at 0.1 mM (%)	*α*-Amylase Inhibition at 0.1 mM (%)	DPPH IC_50_ (μM) *^b^*	ABTS^•^+ IC_50_ (μM) *^b^*
**1**	NA *^c^*	— *^d^*	—	—	—
**2**	NA	NA	10.6 ± 2.3 f	43.17 ± 1.06 d	10.62 ± 0.48 f
**3**	NA	42.3 ± 8.7 bc	NA	23.83 ± 0.89 b	4.13 ± 0.06 c
**4**	6.49 ± 0.27 c	—	—	—	—
**5**	NA	45.1 ± 2.5 b	NA	>250	4.86 ± 0.06 d
**6**	NA	36.5 ± 1.5 c	NA	NA	20.73 ± 0.22 g
**7**	NA	NA	19.9 ± 1.8 d	>250	2.75 ± 0.09 a
**8**	NA	NA	NA	>250	4.17 ± 0.06 c
**9**	5.61 ± 0.44 b	25.4 ± 4.1 d	15.9 ± 3.1 e	29.21 ± 0.37 c	2.68 ± 0.05 a
**10**	NA	19.3 ± 5.6 e	26.1 ± 1.9 c	>250	3.34 ± 0.02 b
**11**	4.55 ± 0.35 a	NA	23.5 ± 1.7 c	>250	3.83 ± 0.05 c
Orlistat *^e^*	4.46 ± 0.13 a				
Acarbose *^e^*		93.2 ± 0.1 a	51.8 ± 2.5 a		
L-(+)-ascorbic acid *^e^*				13.66 ± 0.13 a	10.06 ± 0.19 e

*^a^* Data are recorded as mean ± SD (*n* = 3). Means with the same letter are not significantly different (one-way analysis of variance, *α* = 0.05). *^b^* IC_50_: the ultimate concentration of sample needed to inhibit 50% of enzyme activity or clear away 50% of free radicals. *^c^* NA: no activity. *^d^* It was not measured. *^e^* Positive control.

## Data Availability

The data presented in this study are available in Supplementary materials.

## Supplementary Materials

The following supporting information can be downloaded at: https://www.mdpi.com/article/10.3390/molecules27217390/s1, ^1^H NMR, ^13^C NMR, HMBC, HRESIMS, and IR spectra of compounds **1** (Figure S1) and **4** (Figure S4); ^1^H NMR, ^13^C NMR, ^1^H-^1^H COSY, HSQC, HMBC, HRESIMS and IR spectra of compounds **2** (Figure S2), **3** (Figure S3), **5** (Figure S5), and **6** (Figure S6); determination of bioactivities (S1.); ^1^H NMR and ^13^C NMR data of **1a**, **4a**, and **7**–**11** (S2.).

## Sample Availability

Samples of the compounds are not available from the authors.
