# Supplementary material for: Phenylethanoid and Phenylmethanoid Glycosides from the Leaves of Ligustrum robustum and Their Bioactivities
Source: Molecules. 2022 Oct 31;27(21):7390. doi: 10.3390/molecules27217390 (PMC9657303; doi:10.3390/molecules27217390)
Supplement: Supplementary file 1 [file molecules-27-07390-s001.zip › molecules-1951487-supplementary.pdf]

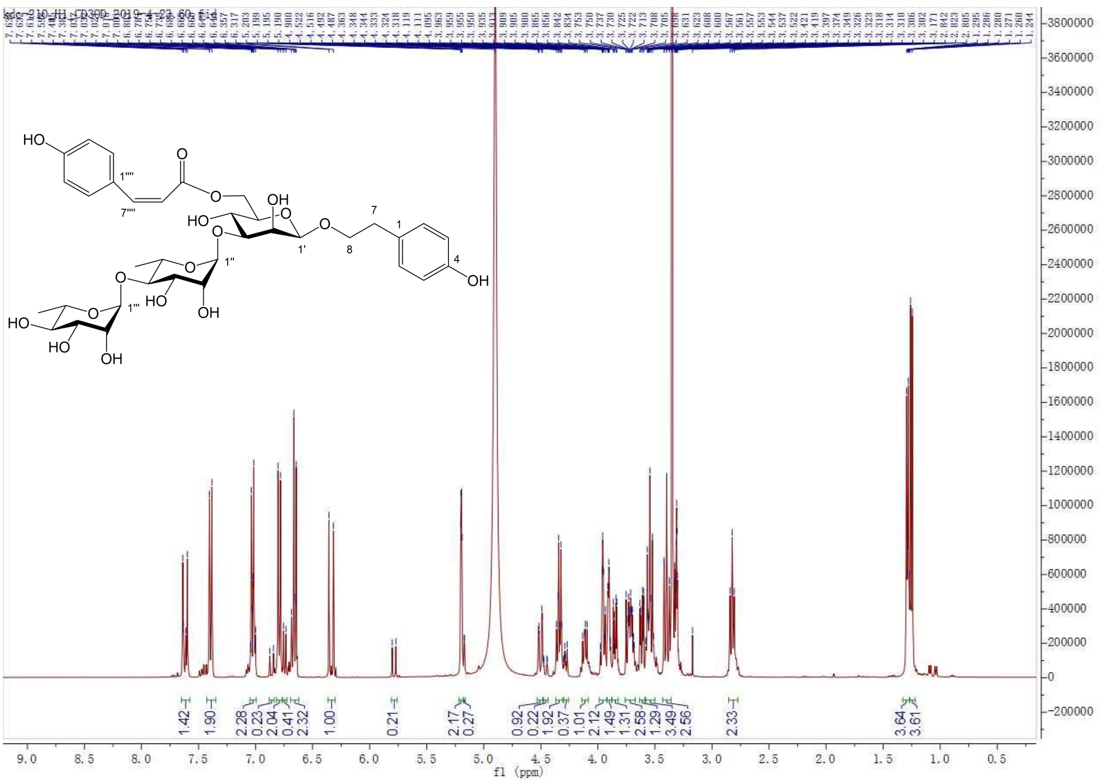

Figure S1-1 ^{1}H NMR spectrum of compound **1** in CD_3OD (400 MHz)

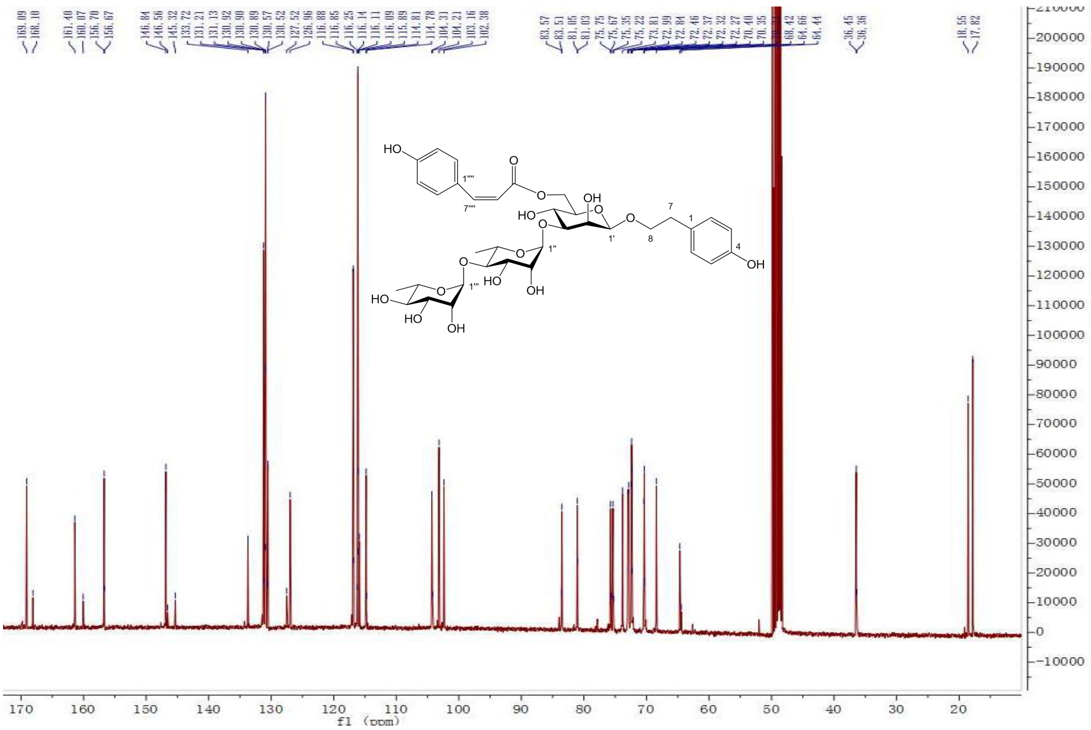

Figure S1-2 ^{13}C NMR spectrum of compound **1** in CD_3OD (100 MHz)

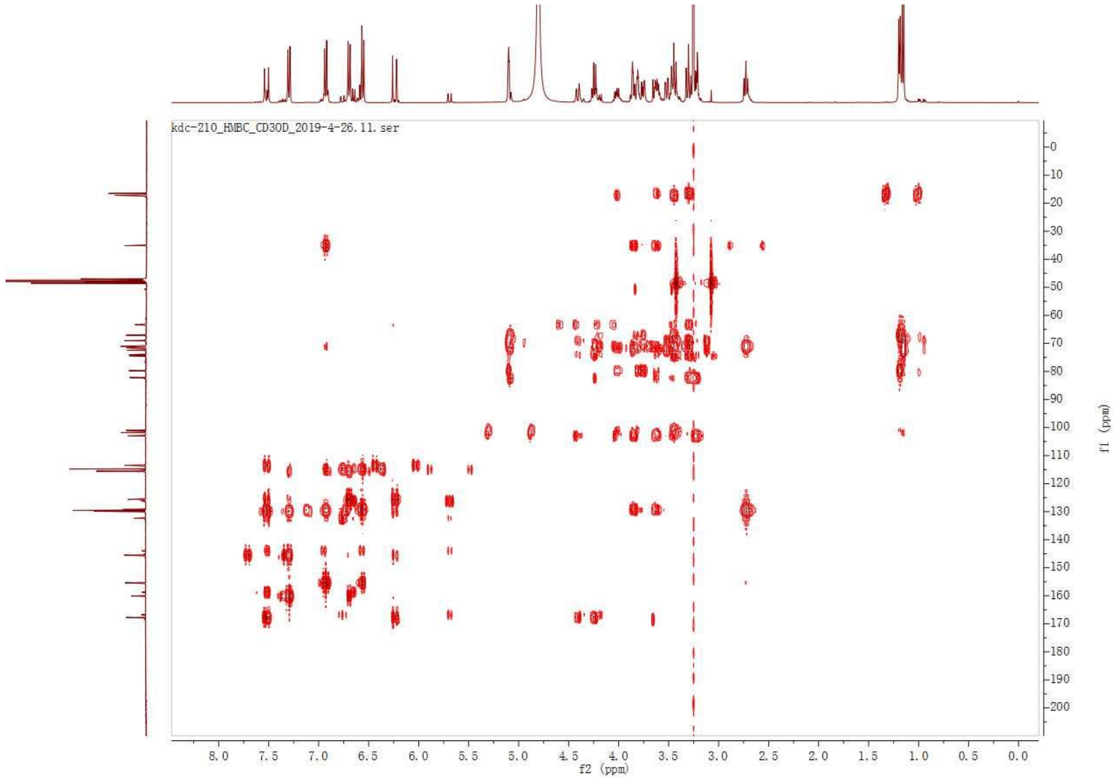

Figure S1-3 HMBC spectrum of compound **1** in CD_3OD (400 MHz)

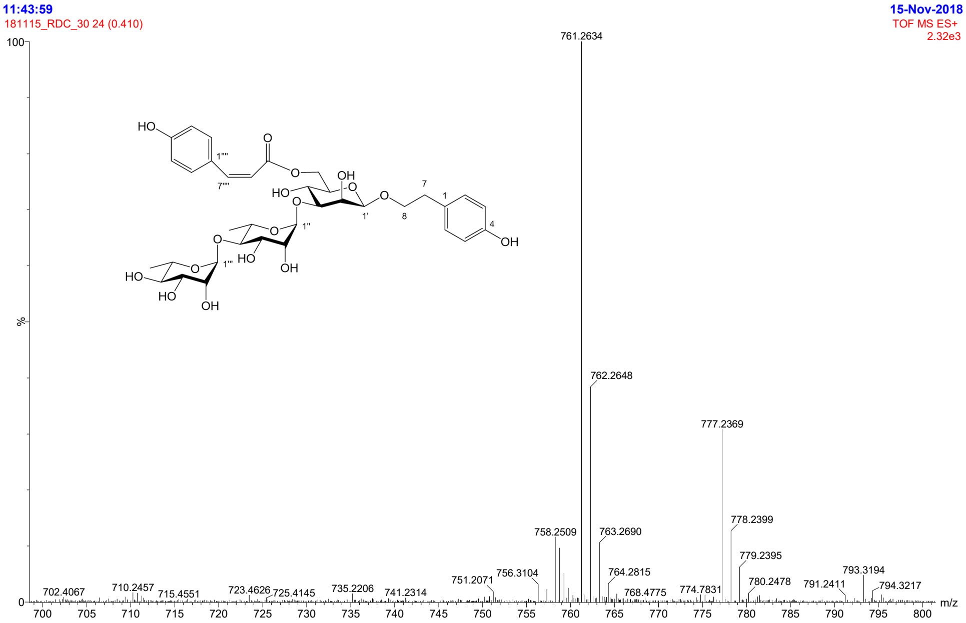

Figure S1-4 HRESIMS spectrum of compound **1**

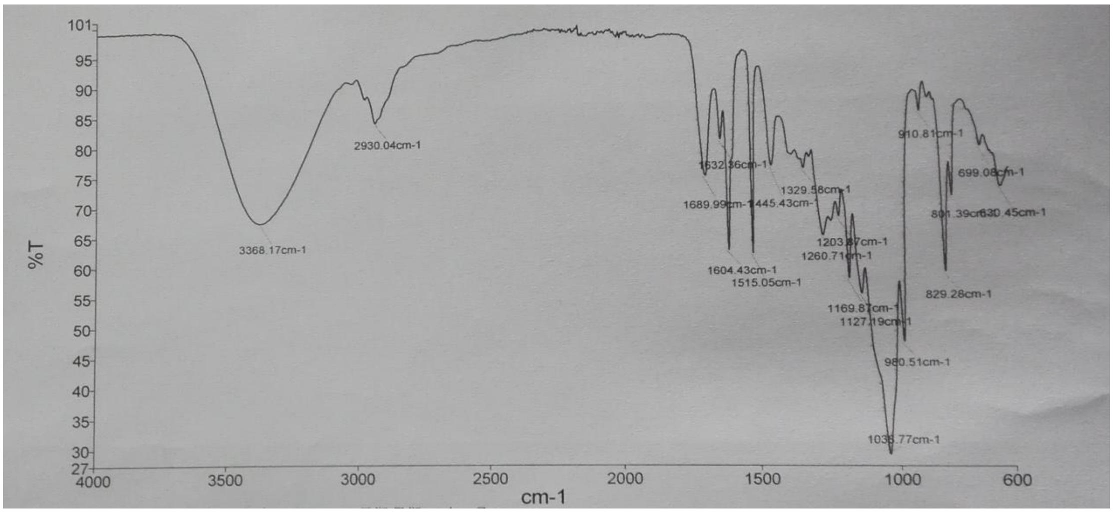

Figure S1-5 IR spectrum of compound **1** (film)

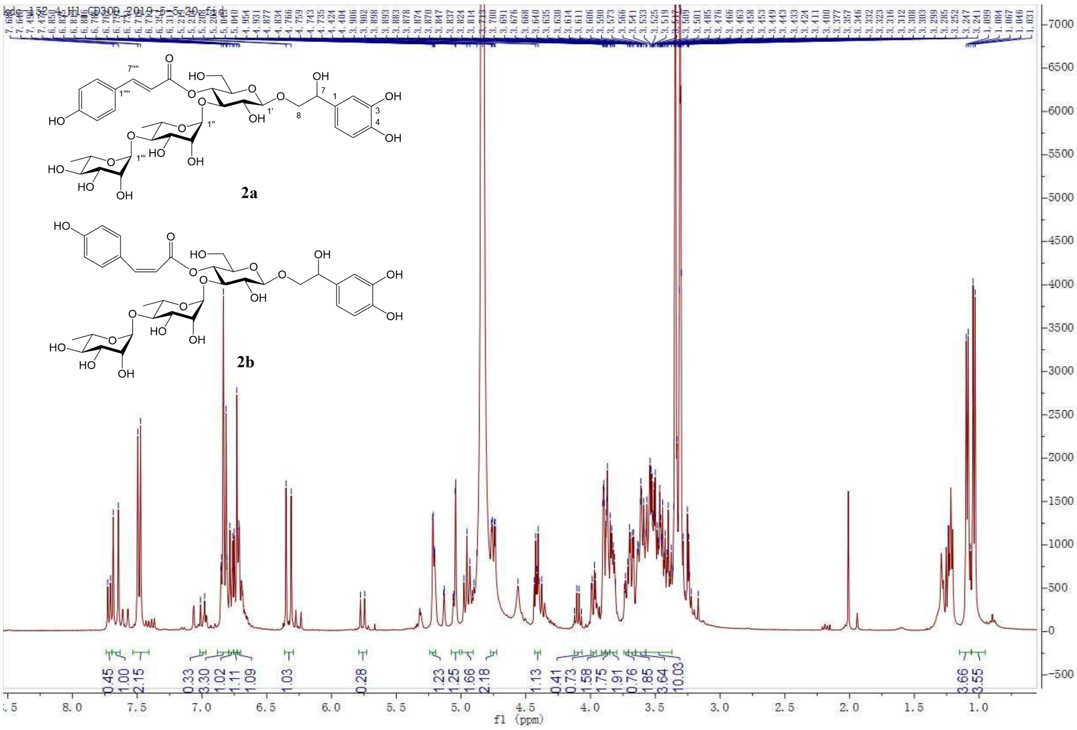

Figure S2-1 ^{1}H NMR spectrum of mixture **2** in CD_3OD (400 MHz)

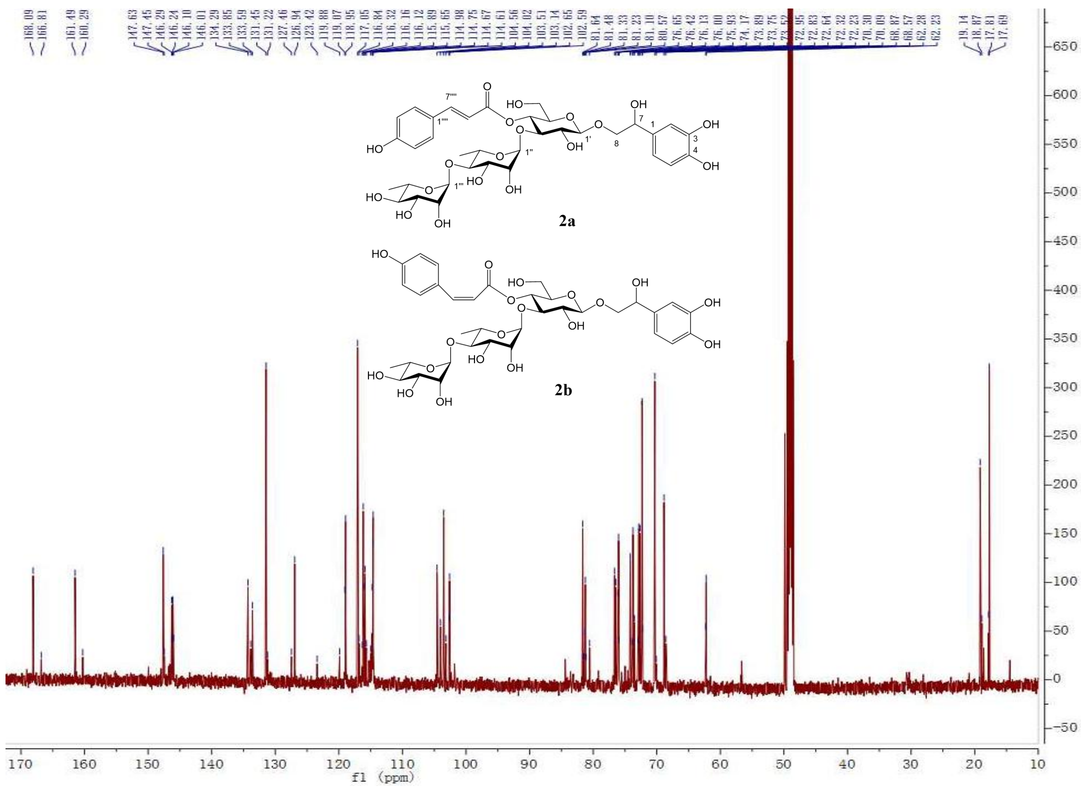

Figure S2-2 ^{13}C NMR spectrum of mixture **2** in CD_3OD (150 MHz)

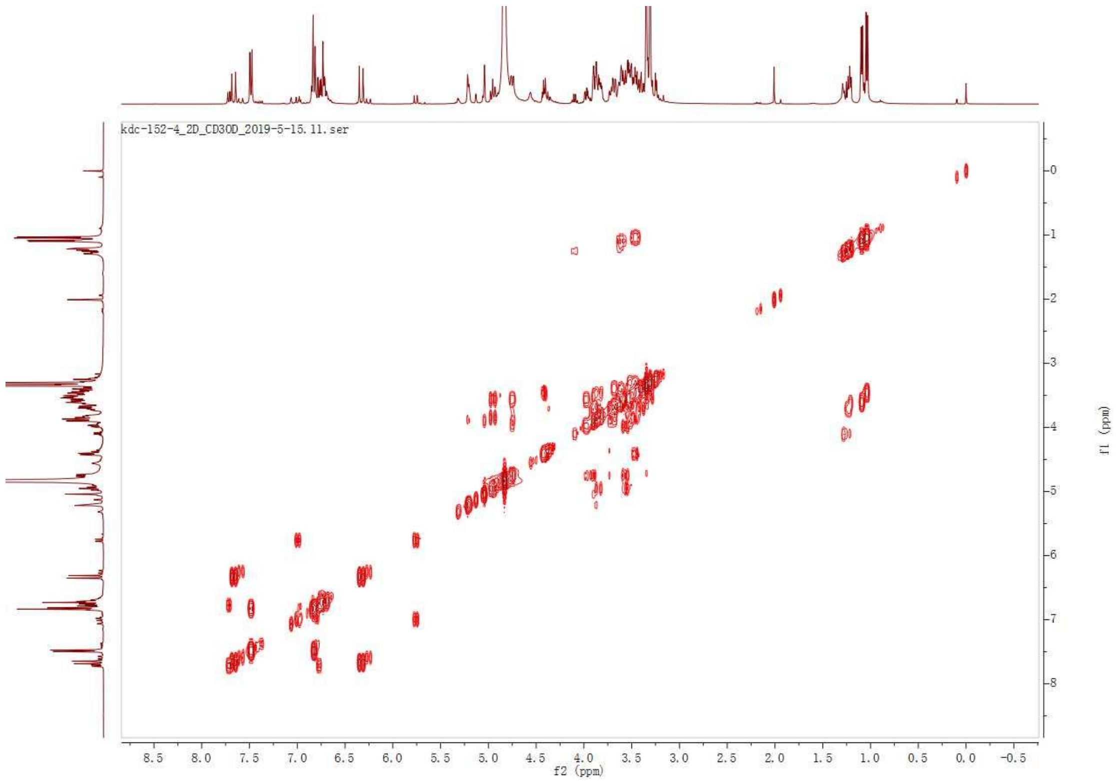

Figure S2-3 ^{1}H-^{1}H COSY spectrum of mixture **2** in CD_3OD (400 MHz)

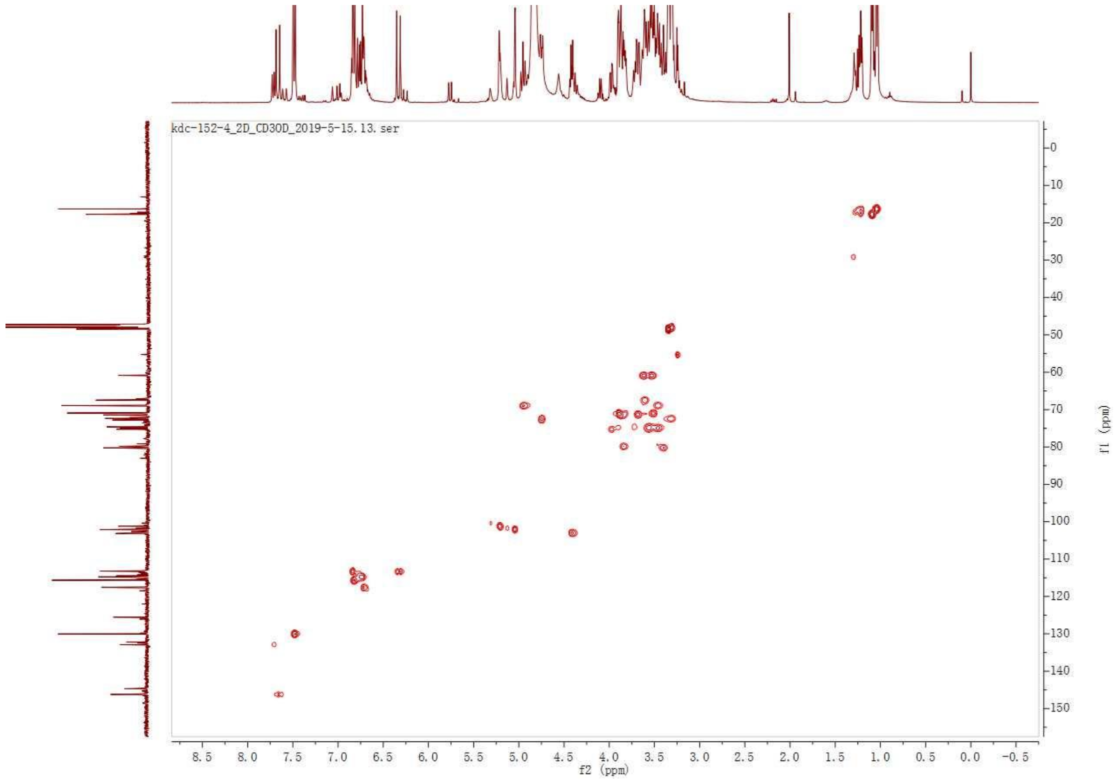

Figure S2-4 HSQC spectrum of mixture **2** in CD_3OD (400 MHz)

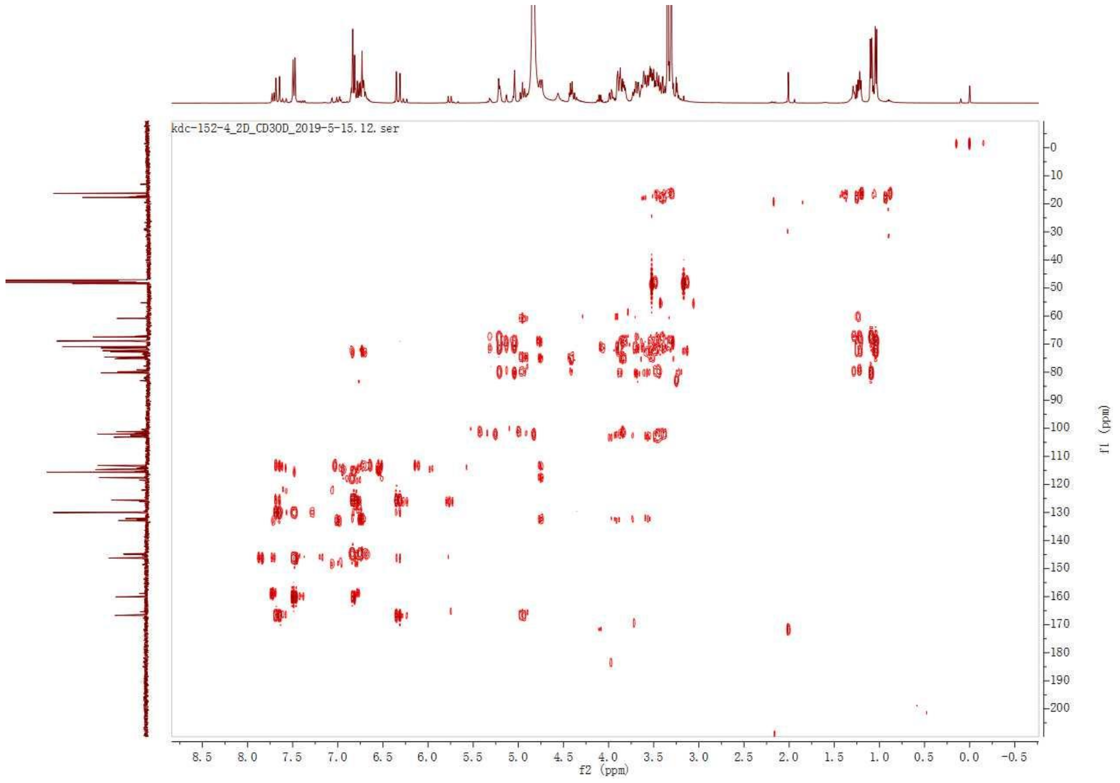

Figure S2-5 HMBC spectrum of mixture **2** in CD_3OD (400 MHz)

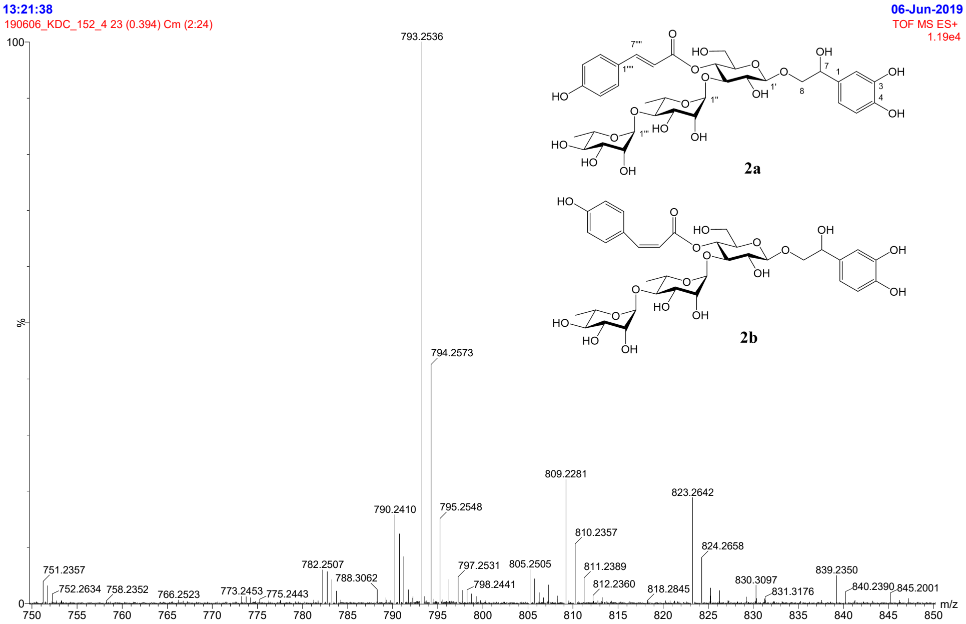

Figure S2-6 HRESIMS spectrum of mixture **2**

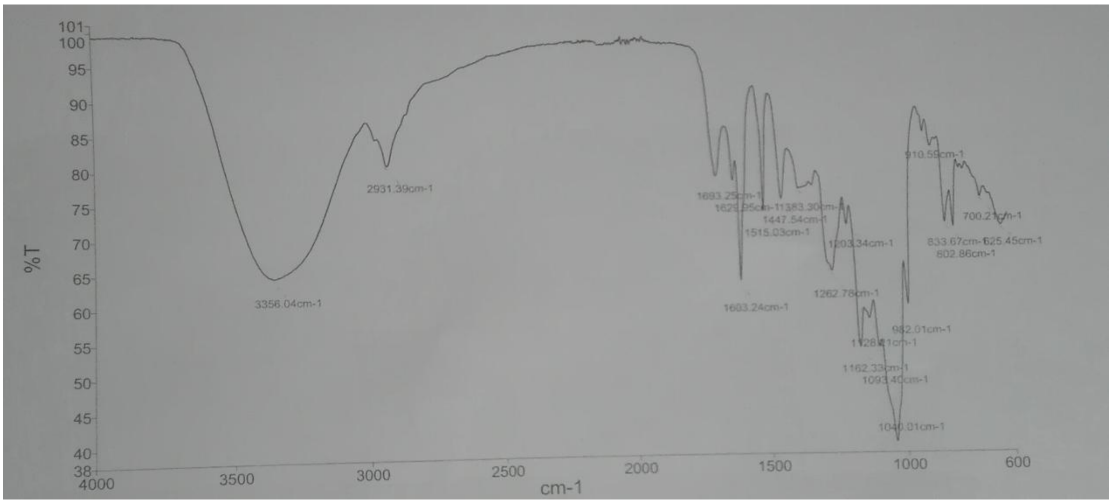

Figure S2-7 IR spectrum of mixture **2** (film)

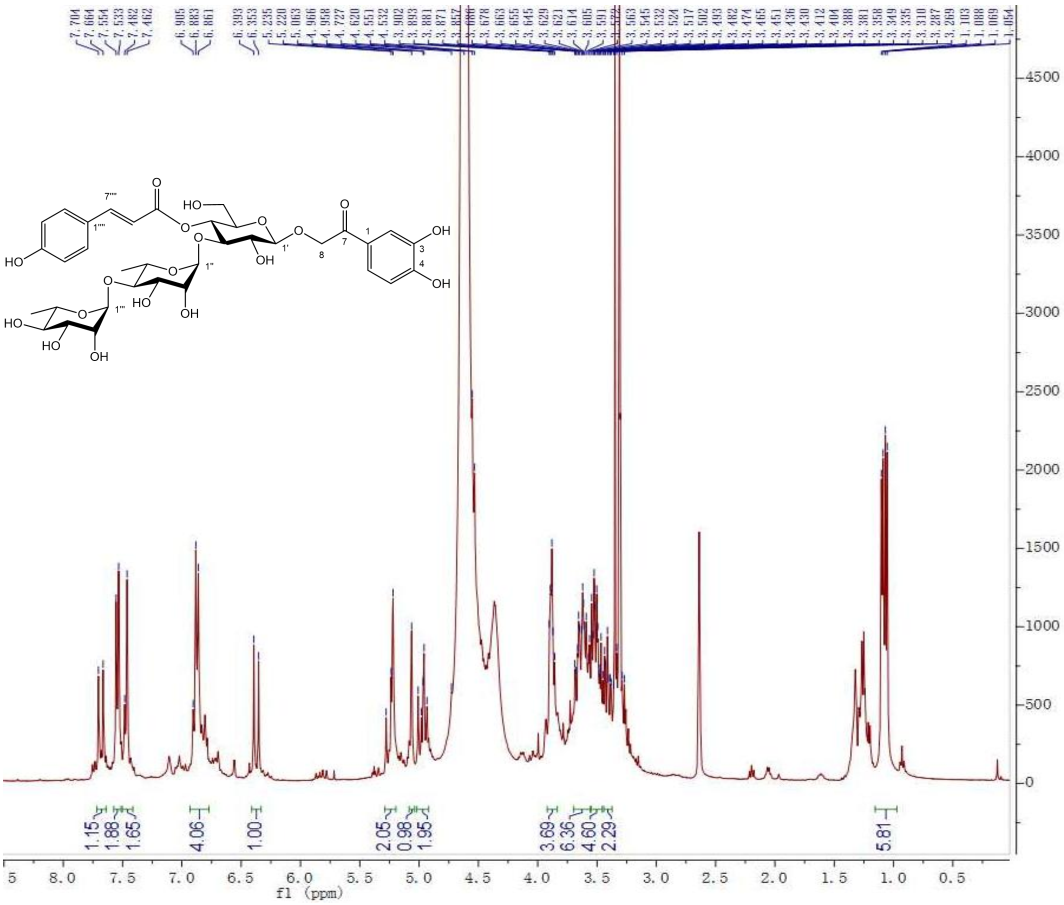

Figure S3-1 ^{1}H NMR spectrum of compound **3** in (CD_3OD+DMSO-d_6) (400 MHz)

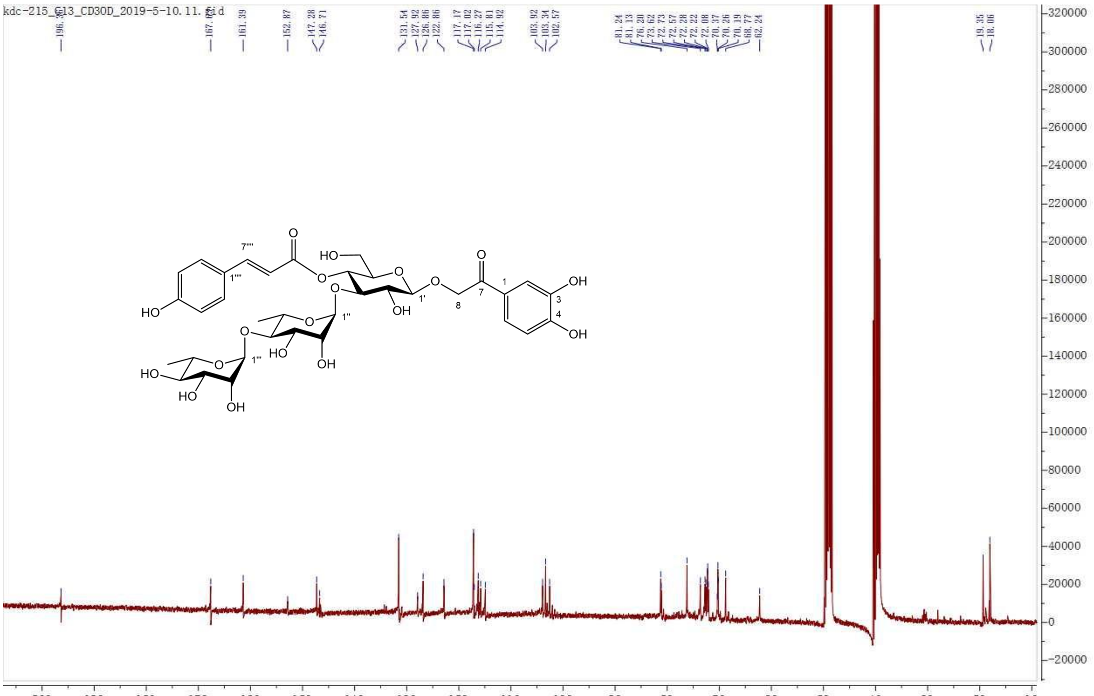

Figure S3-2 ^{13}C NMR spectrum of compound **3** in (CD_3OD+DMSO-d_6) (100 MHz)

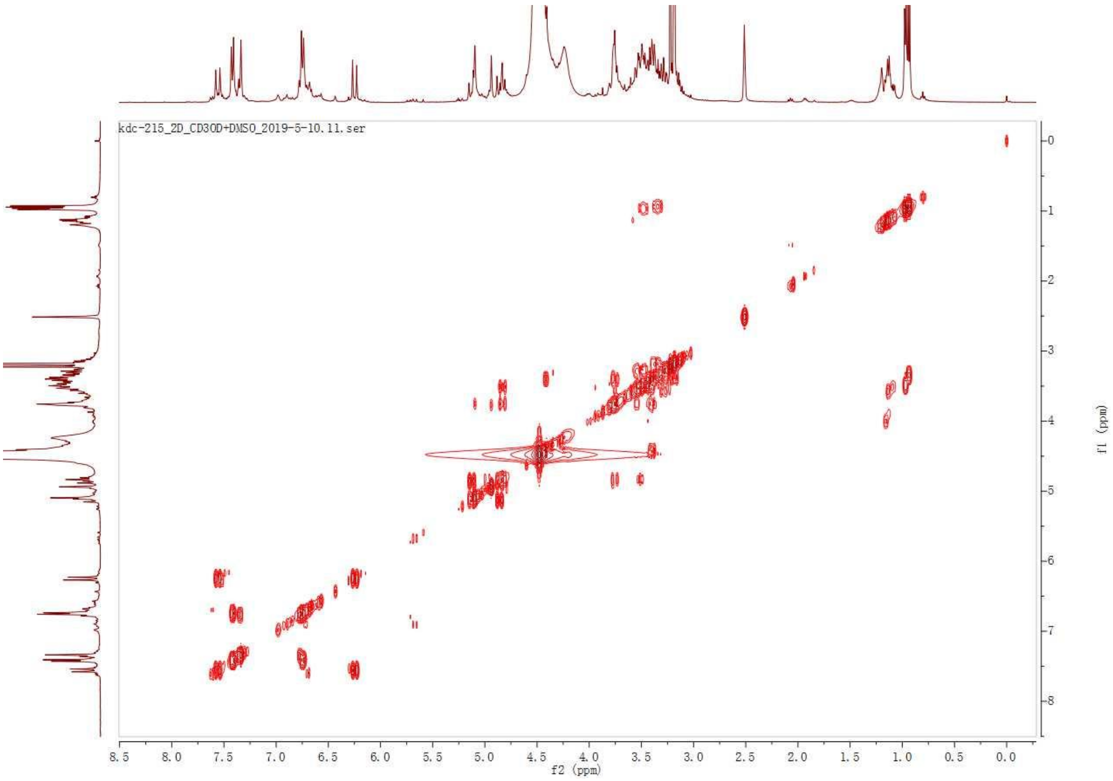

Figure S3-3 ^{1}H-^{1}H COSY spectrum of compound **3** in (CD_3OD+DMSO-d_6) (400 MHz)

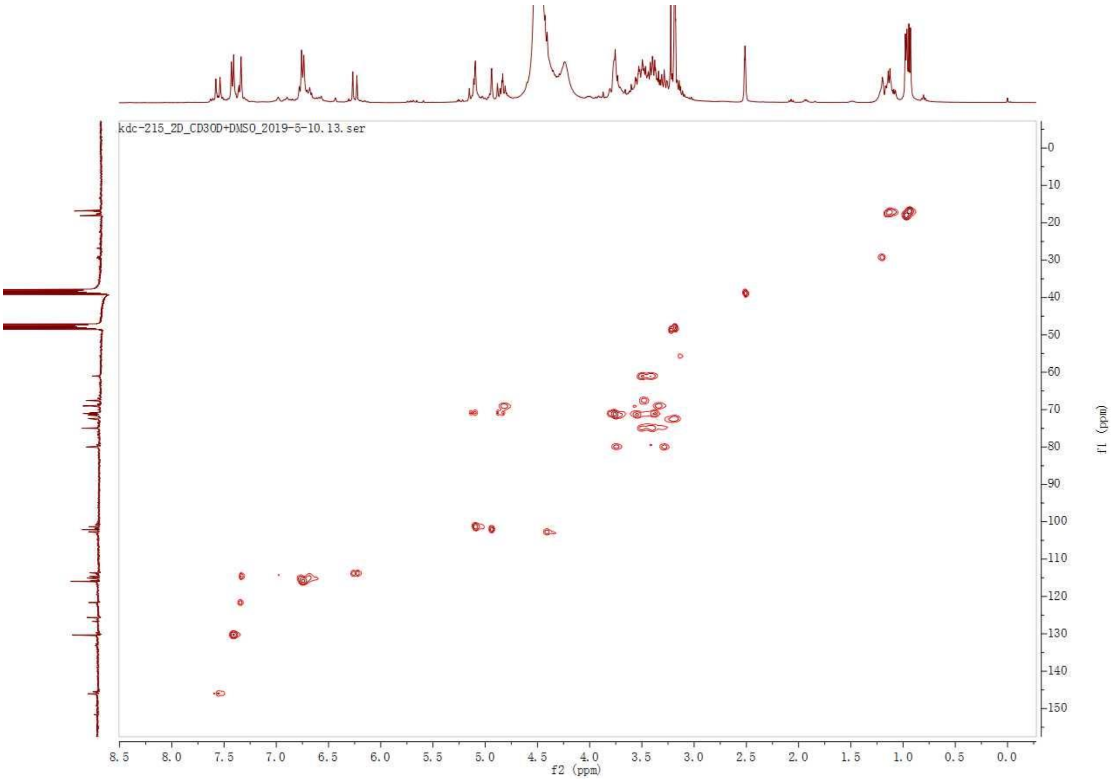

Figure S3-4 HSQC spectrum of compound **3** in (CD_3OD+DMSO-d_6) (400 MHz)

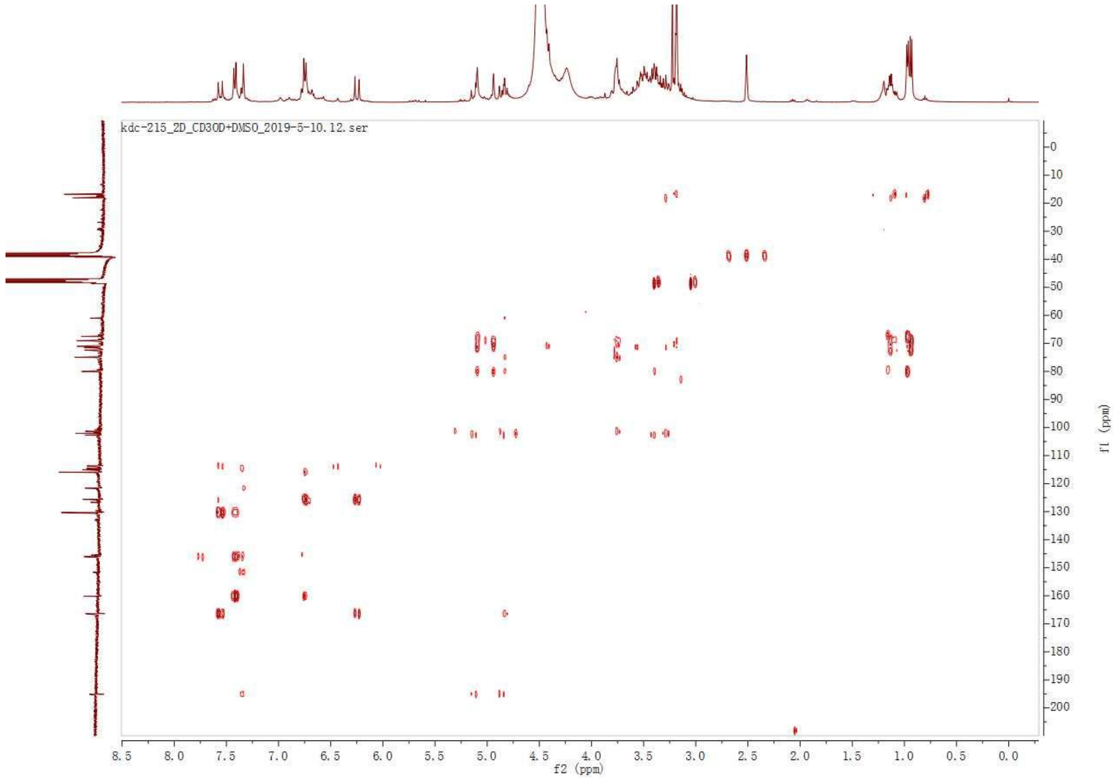

Figure S3-5 HMBC spectrum of compound **3** in (CD_3OD+DMSO-d_6) (400 MHz)

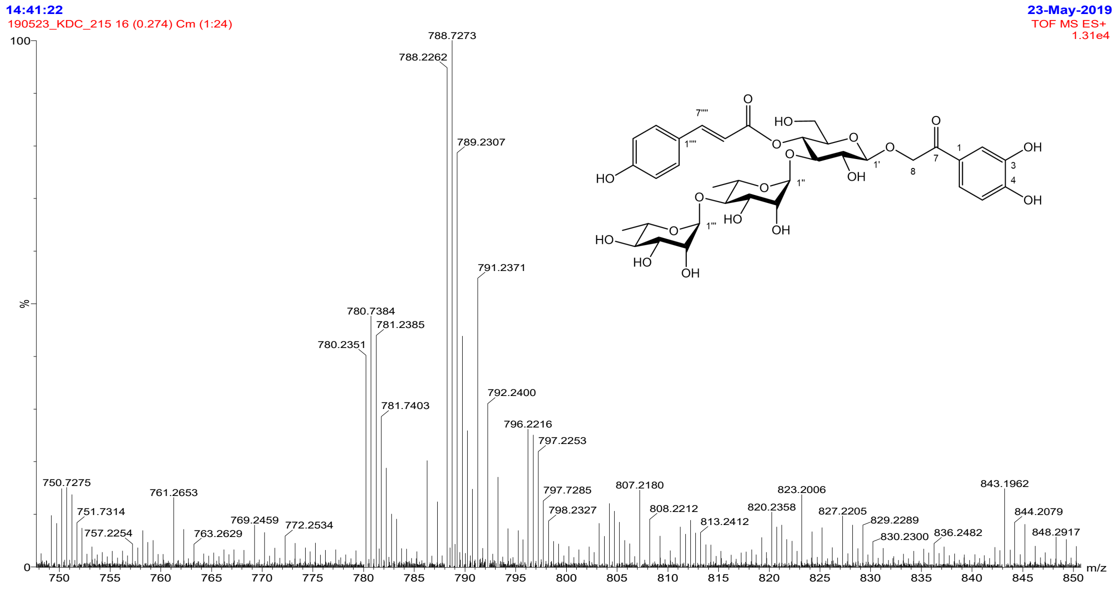

Figure S3-6 HRESIMS spectrum of compound **3**

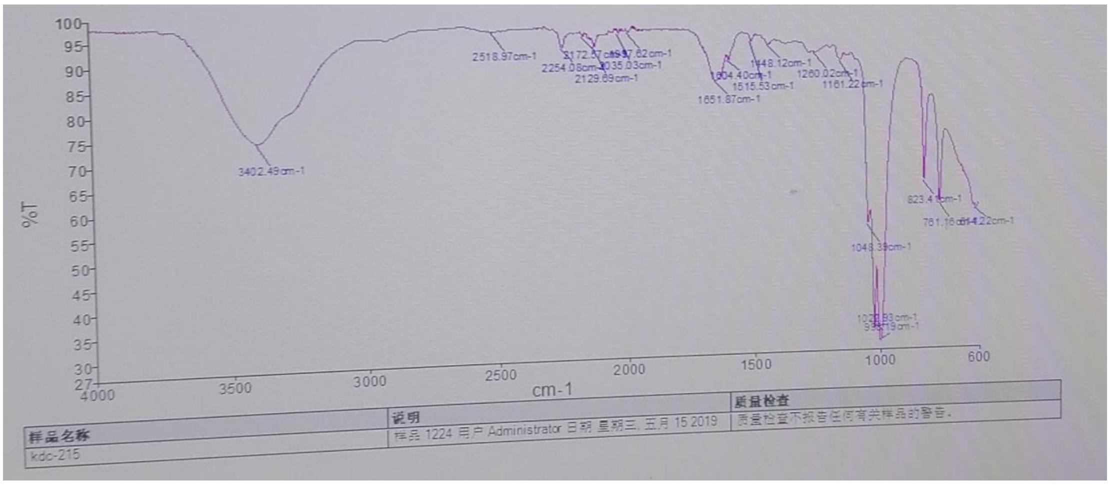

Figure S3-7 IR spectrum of compound **3** (film)

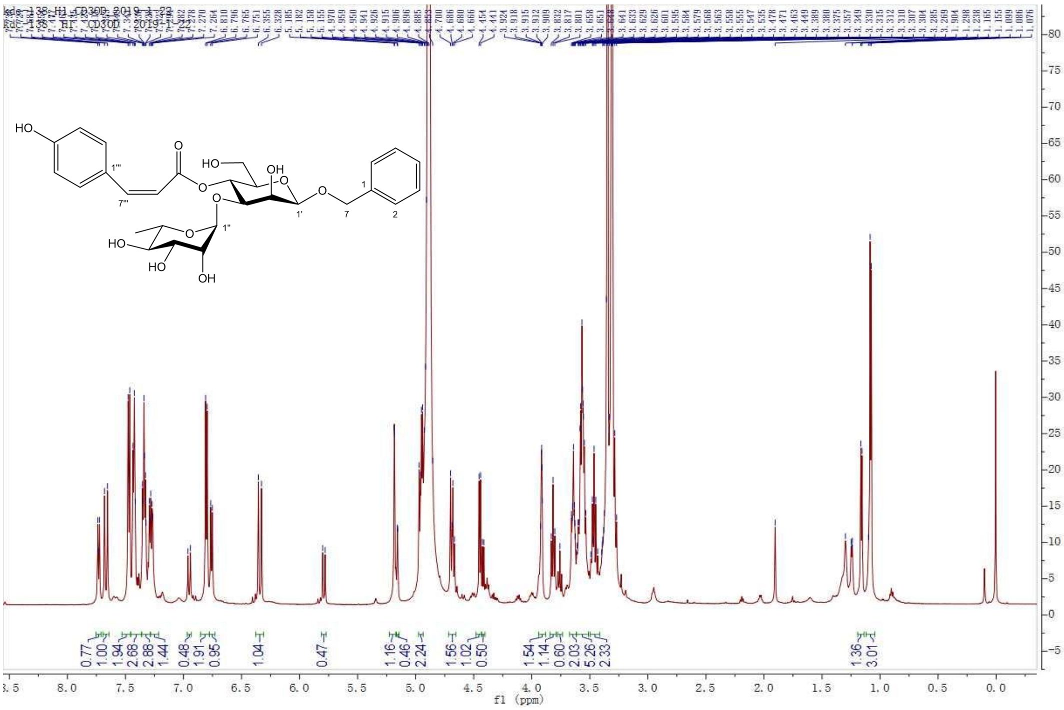

Figure S4-1 ^{1}H NMR spectrum of compound **4** in CD_3OD (600 MHz)

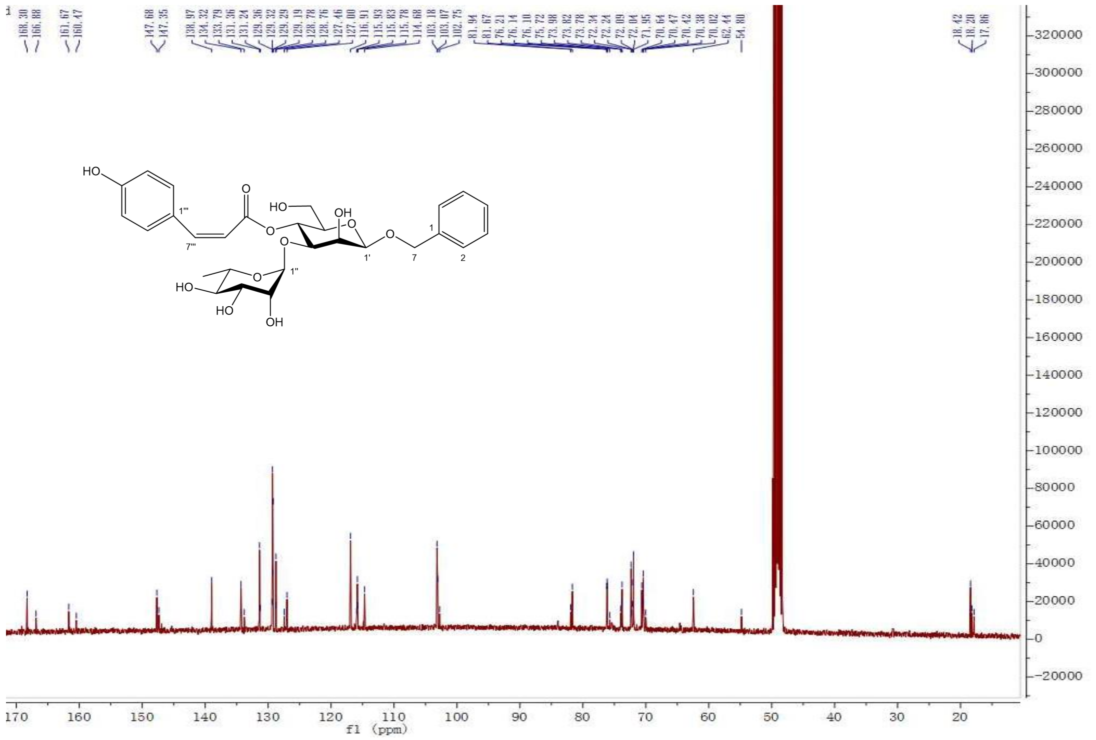

Figure S4-2 ^{13}C NMR spectrum of compound **4** in CD_3OD (100 MHz)

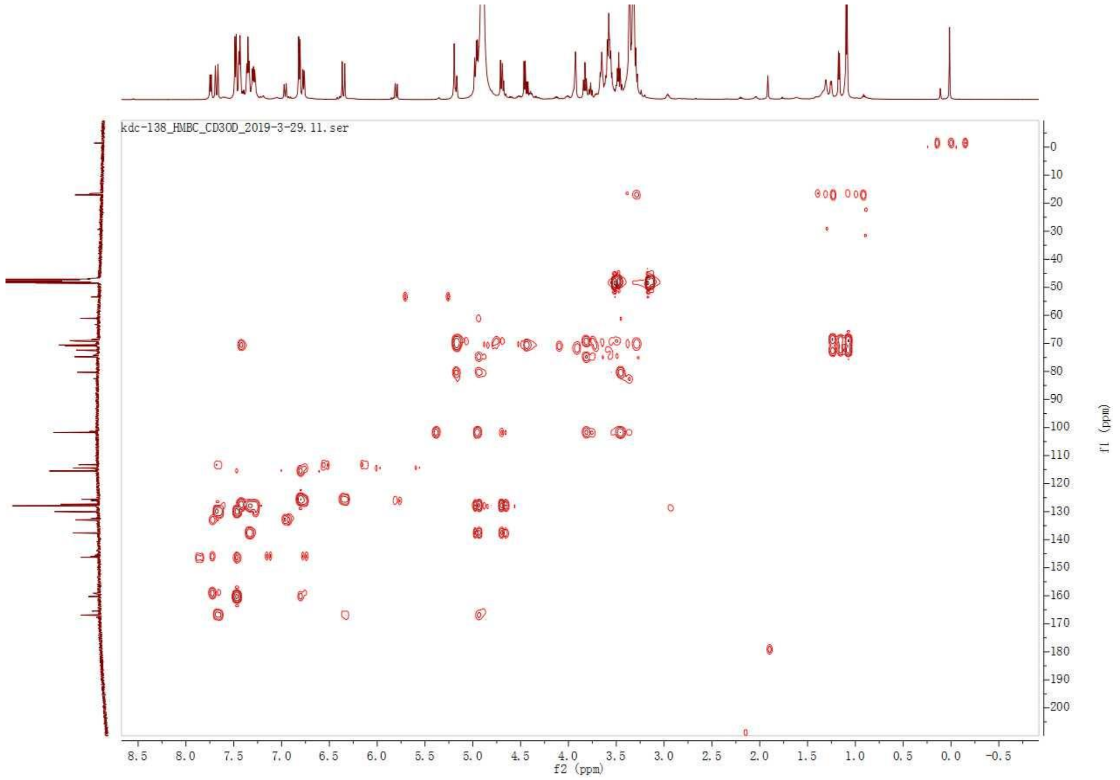

Figure S4-3 HMBC spectrum of compound **4** in CD_3OD (400 MHz)

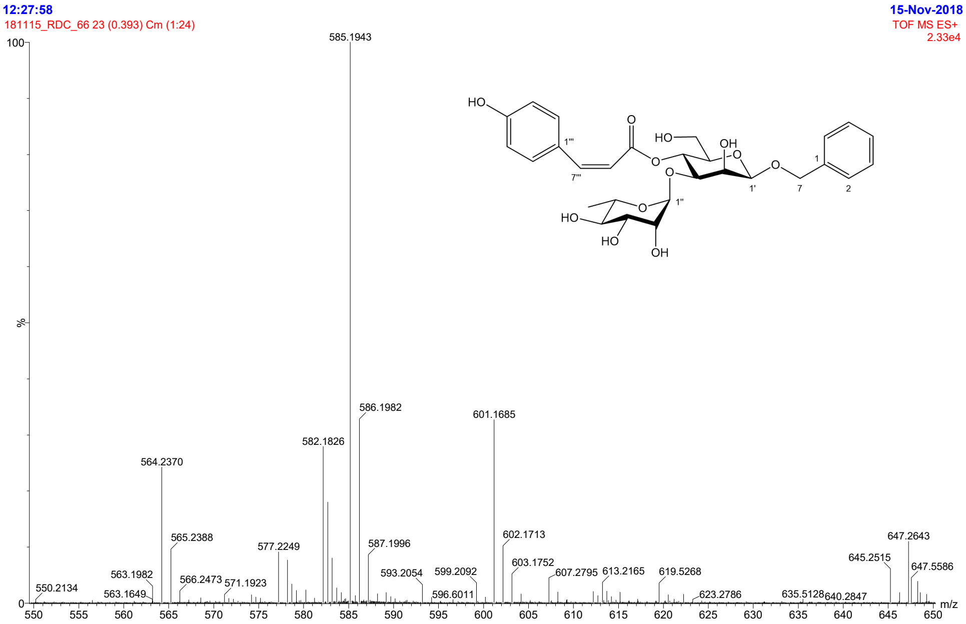

Figure S4-4 HRESIMS spectrum of compound **4**

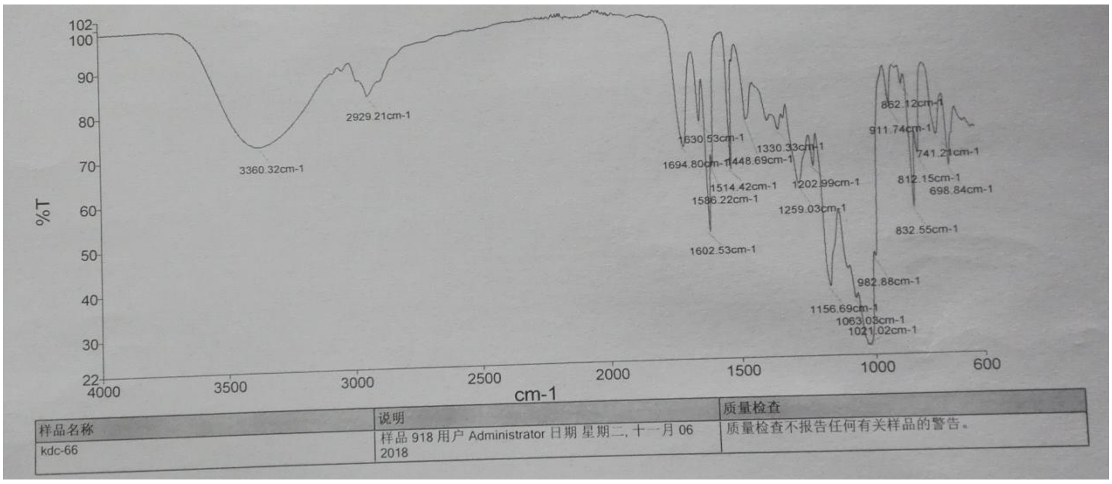

Figure S4-5 IR spectrum of compound **4** (film)

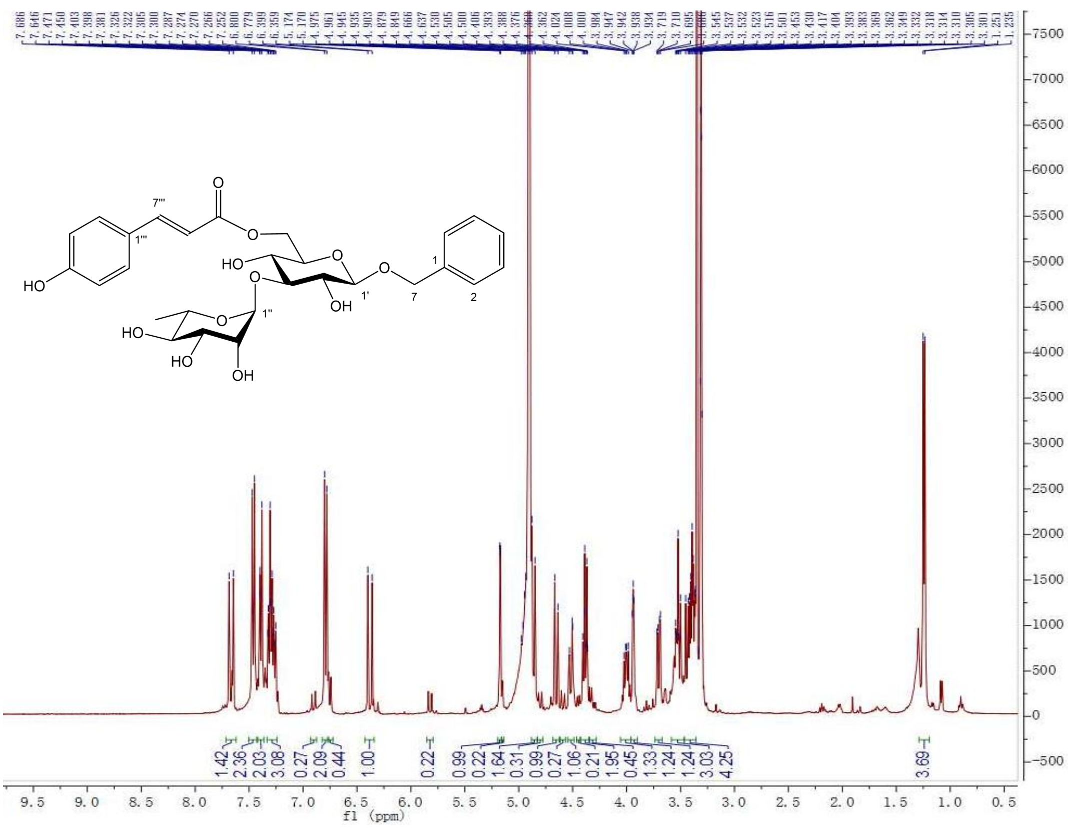

Figure S5-1 ^{1}H NMR spectrum of compound **5** in CD_3OD (400 MHz)

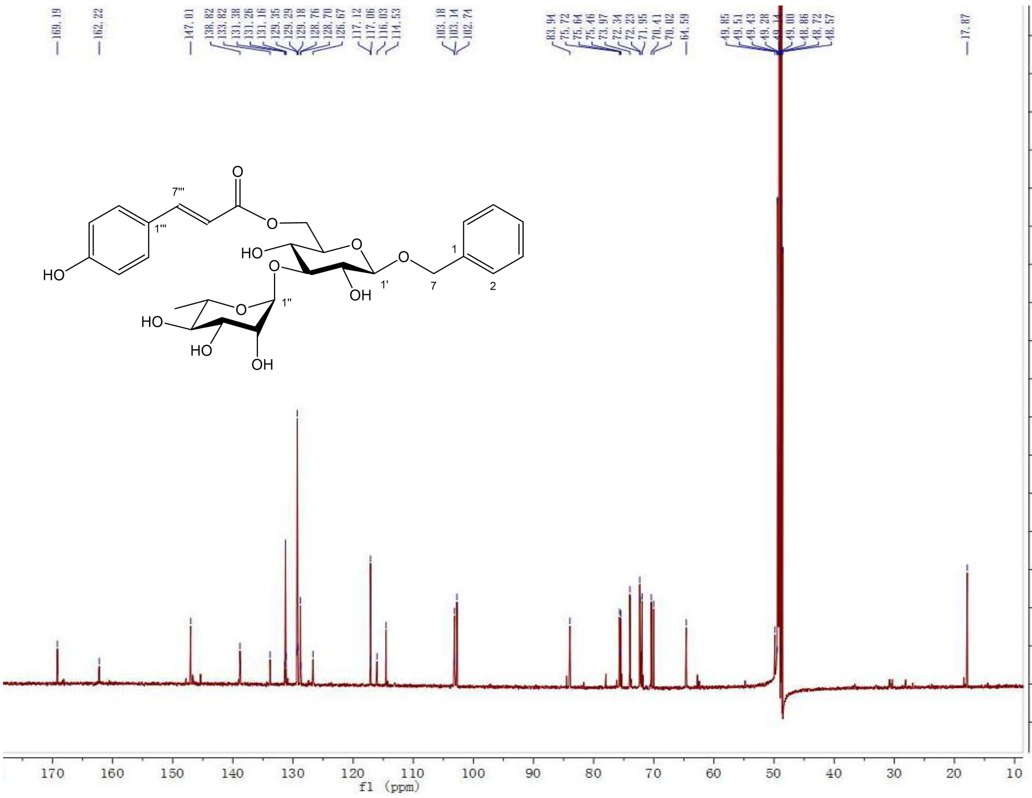

Figure S5-2 ^{13}C NMR spectrum of compound **5** in CD_3OD (150 MHz)

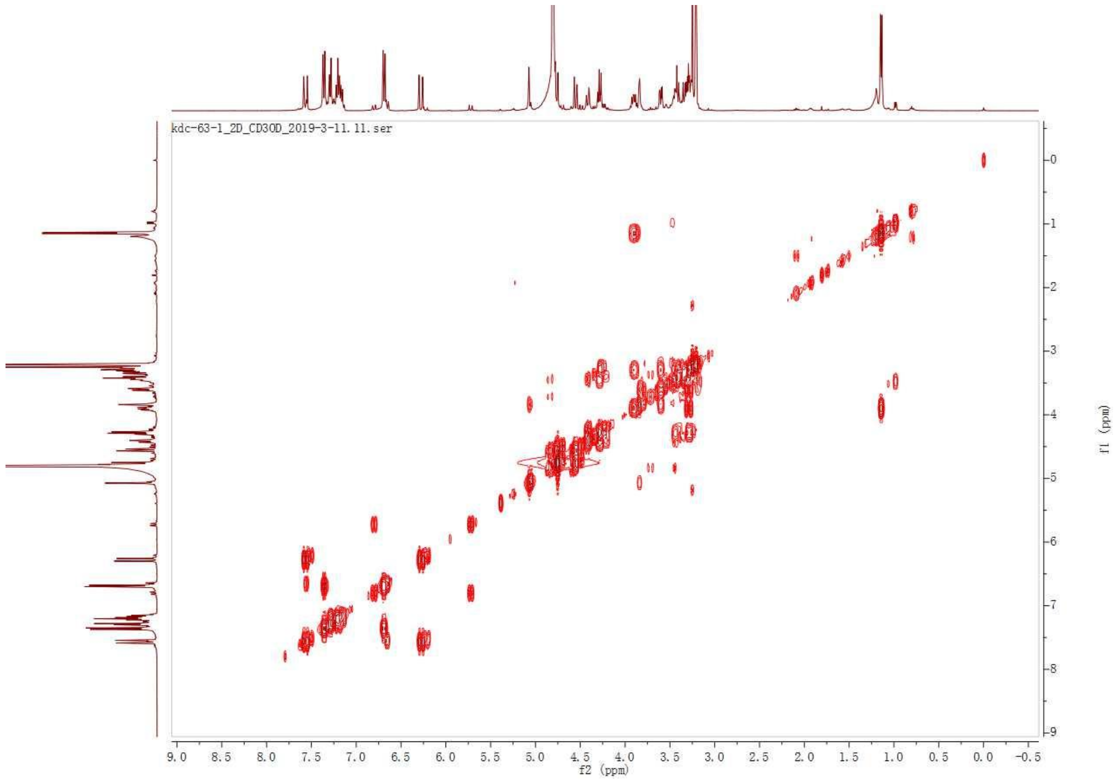

Figure S5-3 ^{1}H-^{1}H COSY spectrum of compound **5** in CD_3OD (400 MHz)

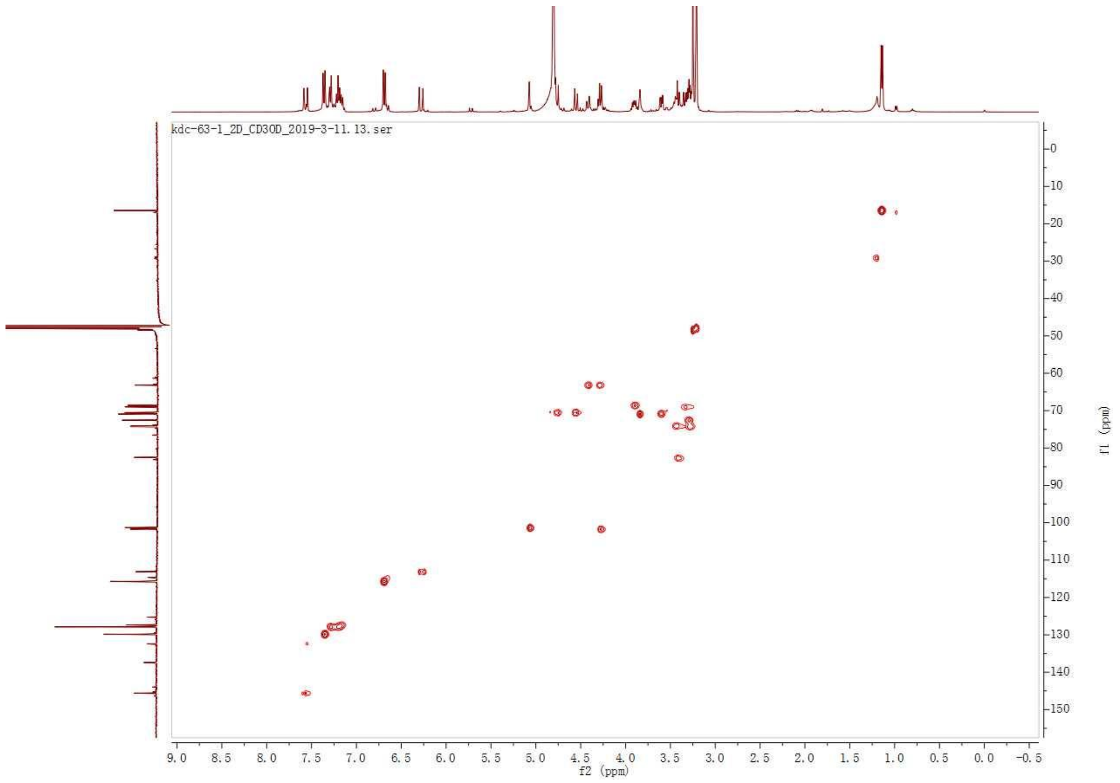

Figure S5-4 HSQC spectrum of compound **5** in CD_3OD (400 MHz)

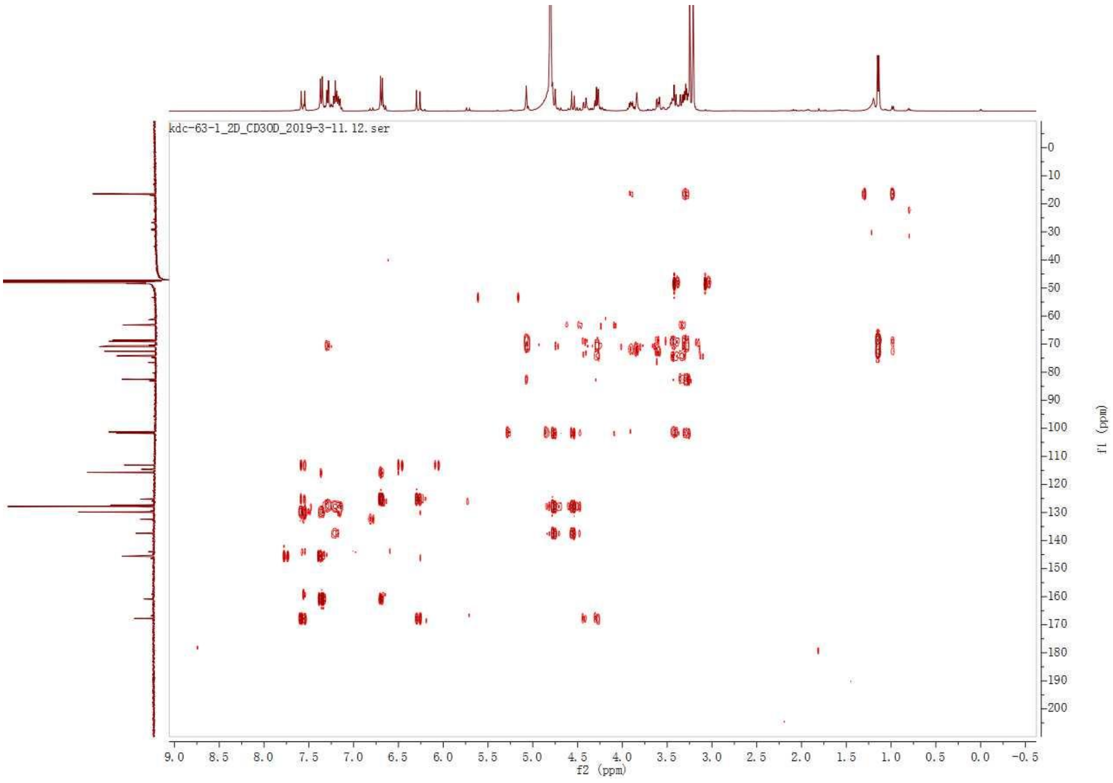

Figure S5-5 HMBC spectrum of compound **5** in CD_3OD (400 MHz)

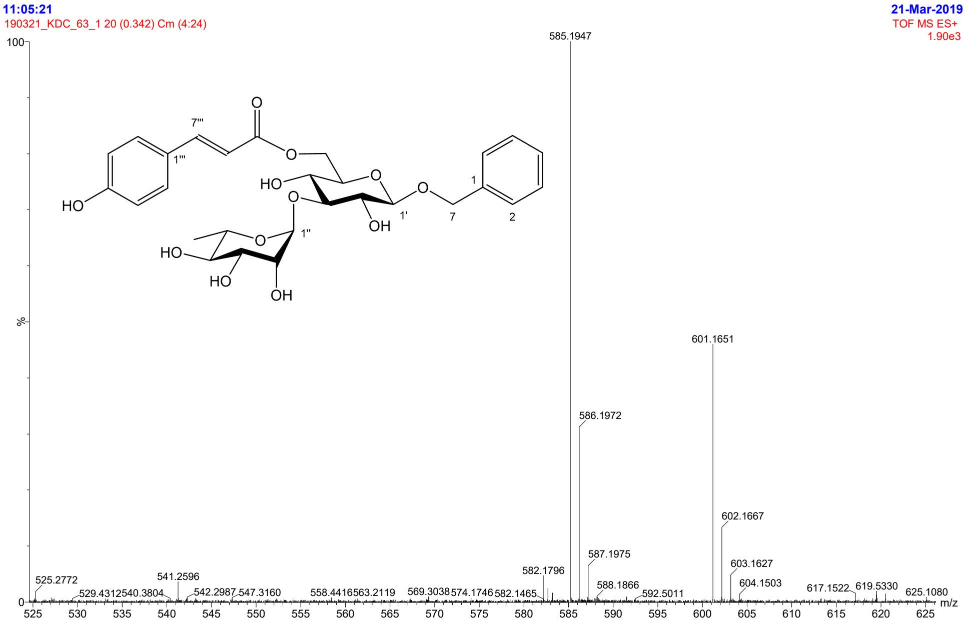

Figure S5-6 HRESIMS spectrum of compound **5**

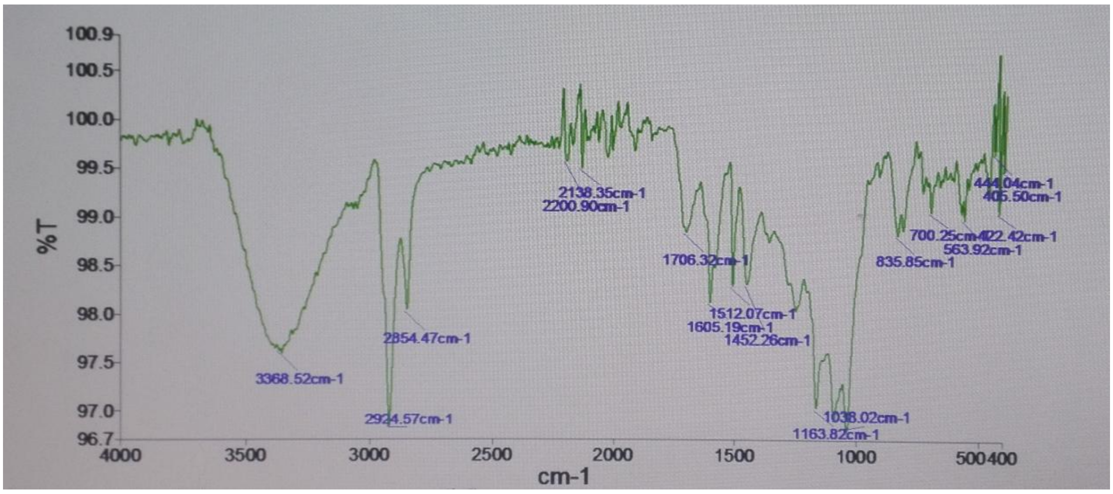

Figure S5-7 IR spectrum of compound **5** (film)

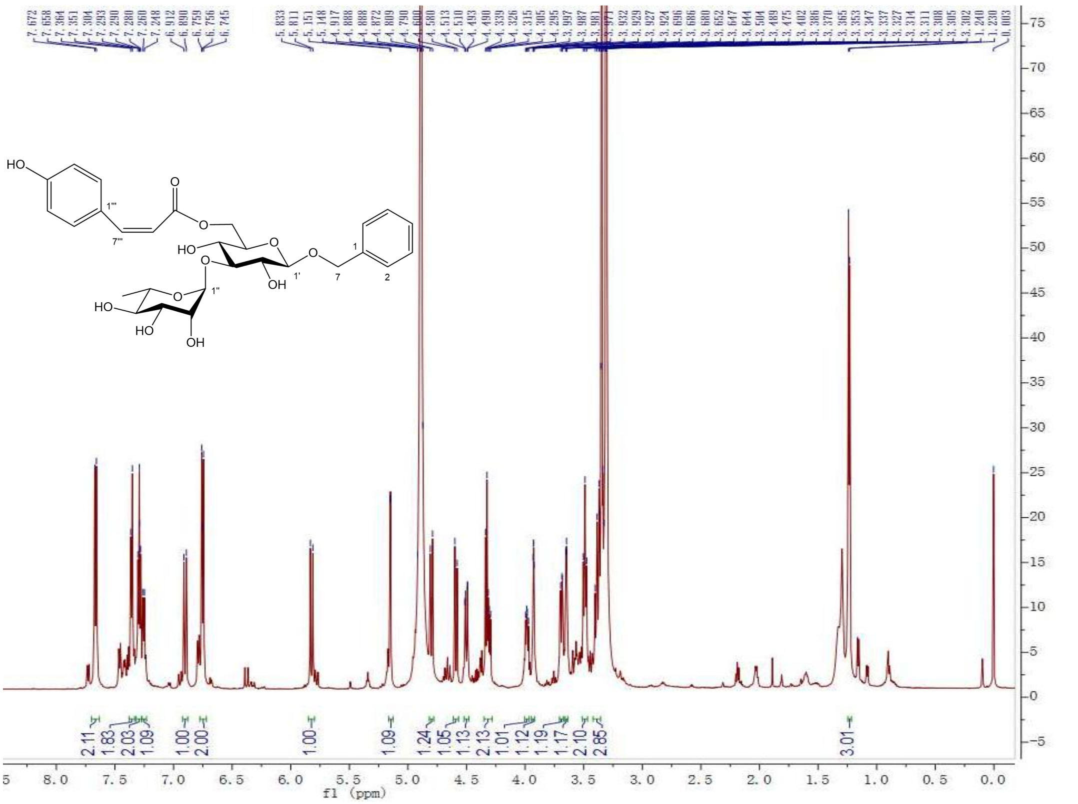

Figure S6-1 ^{1}H NMR spectrum of compound **6** in CD_3OD (600 MHz)

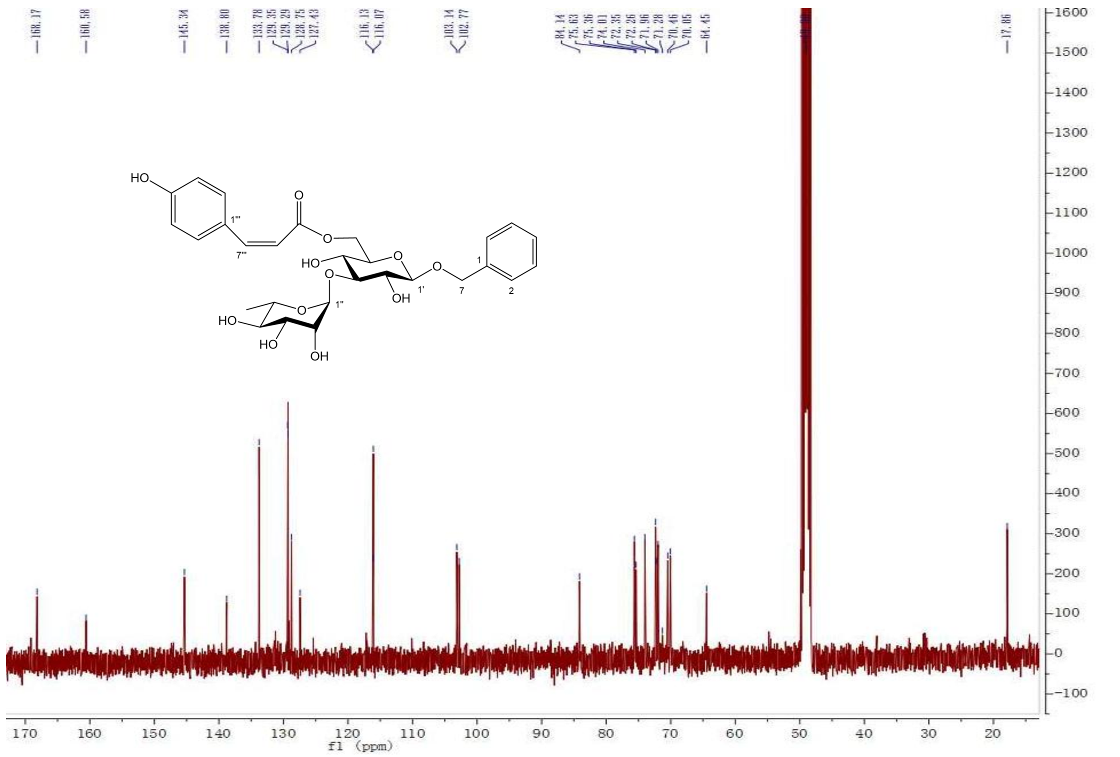

Figure S6-2 ^{13}C NMR spectrum of compound **6** in CD_3OD (100 MHz)

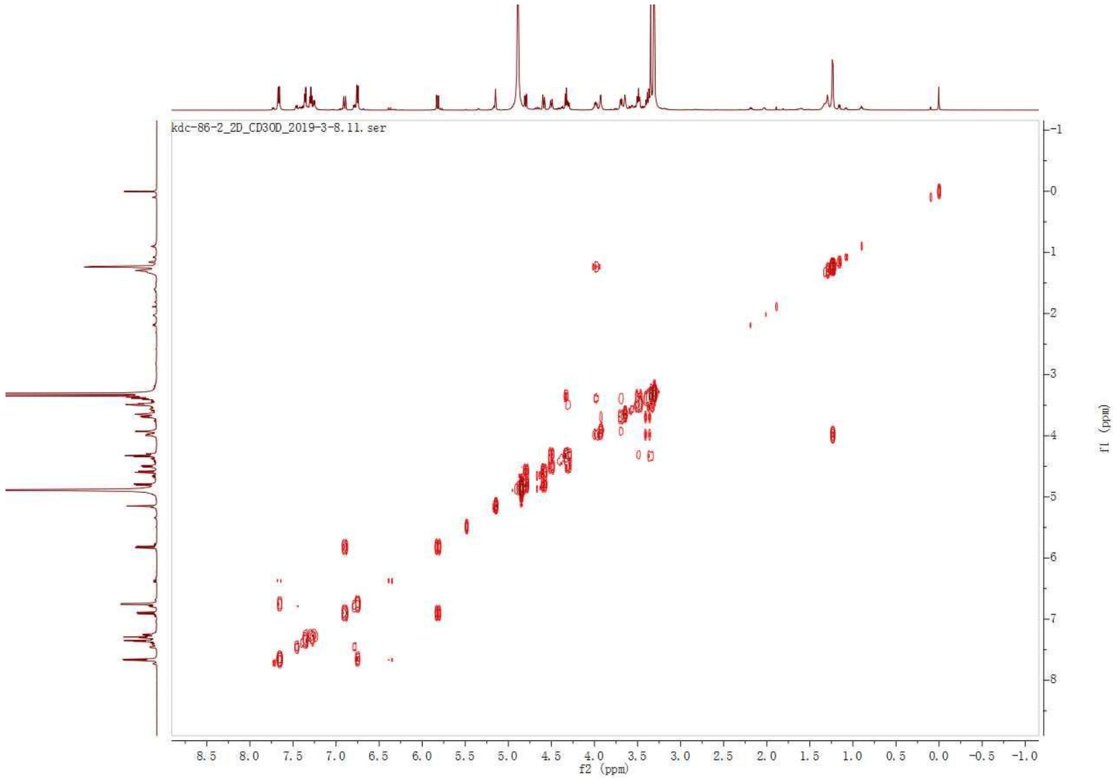

Figure S6-3 ^{1}H-^{1}H NMR spectrum of compound **6** in CD_3OD (400 MHz)

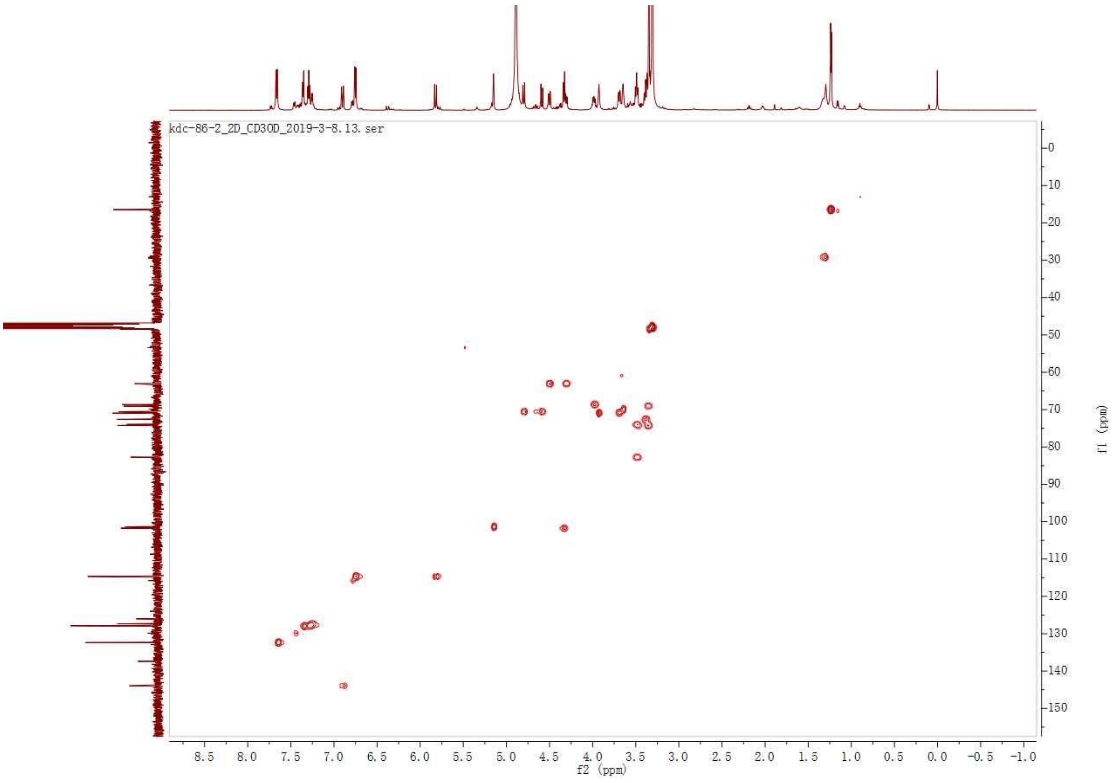

Figure S6-4 HSQC spectrum of compound **6** in CD_3OD (400 MHz)

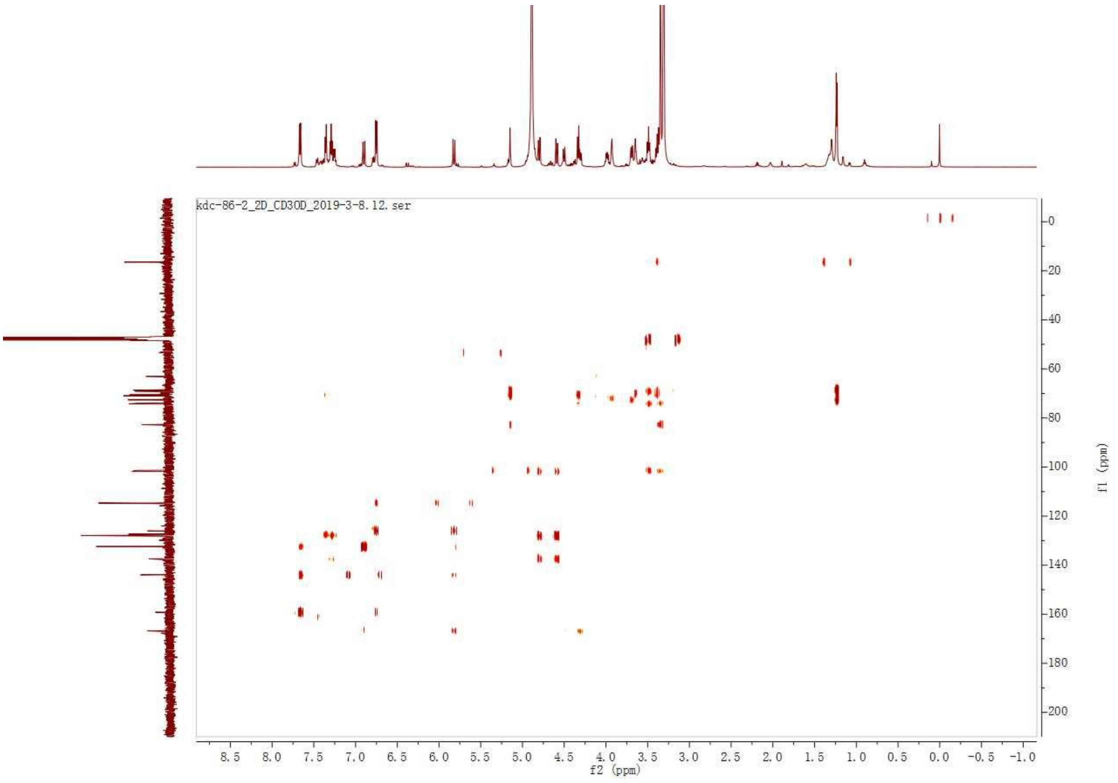

Figure S6-5 HMBC spectrum of compound **6** in CD_3OD (400 MHz)

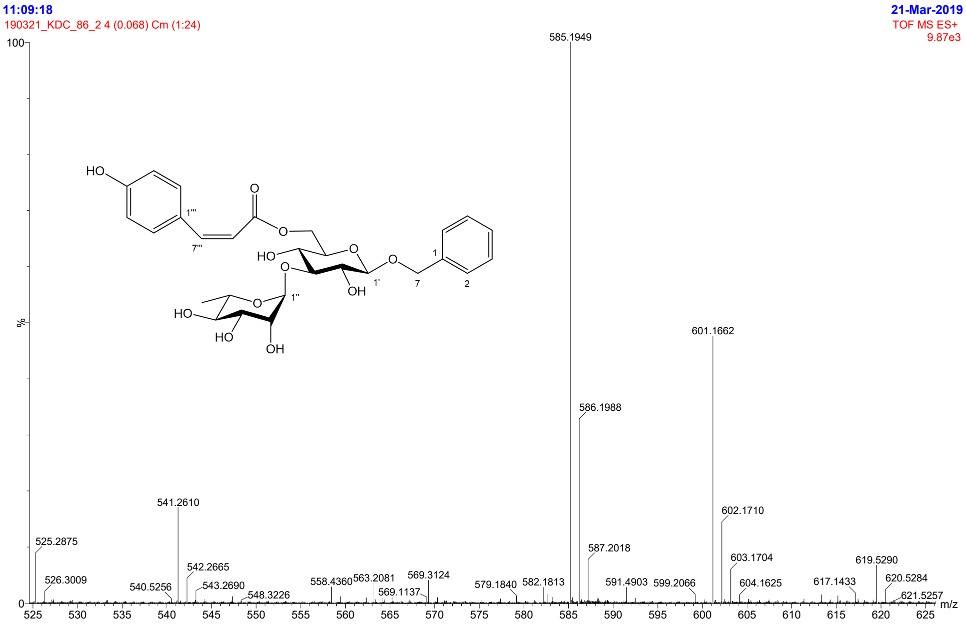

Figure S6-6 HRESIMS spectrum of compound **6**

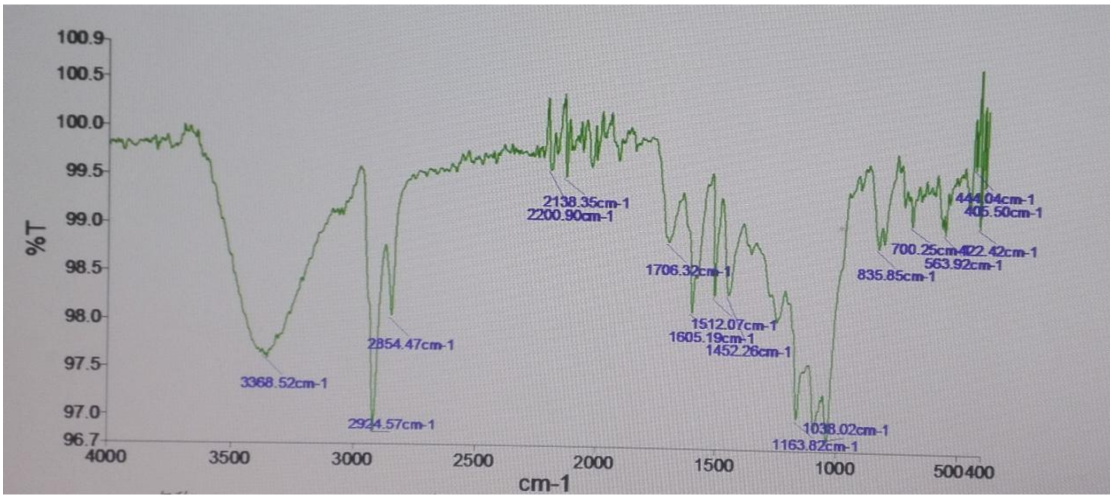

Figure S6-7 IR spectrum of compound **6** (film)

S1. Determination of bioactivities

S1.1. Determination of FAS inhibitory activity

Compounds **1-11** (1.0-1.7 mg) were dissolved in DMSO (100 μL) and then diluted with potassium phosphate buffer (0.1 M, pH 7.0). Sample solution (100 μL, 20-2000 μM, 37 °C) and FAS substrates (1.8 mL, 37 °C) were mixed in a cuvette, and then FAS solution (100 μL, 37 °C, isolated from chicken liver and kept in ice-bath before use) was added. The absorbance of reaction mixture was monitored by a UV-vis spectrophotometer at 340 nm in 1 min. The inhibitory effect was calculated by the following equation: FAS inhibition (%) = $(A_{\text{control}} - A_{\text{sample}})/A_{\text{control}} \times 100\%$, where A_{control} represented the FAS activity in the control group (phosphate buffer instead of sample solution), A_{sample} represented the FAS activity in the sample groups. The FAS activity was calculated as $(A_0 - A_1)$/1 min, in which A_0 was the absorbance of the reaction mixture when the FAS was added, and A_1 was the absorbance of the reaction mixture after reaction 1 min. Orlistat was used as the positive control.

FAS substrates: 0.1 M potassium phosphate buffer (pH 7.0), 1 mM ethylene-diaminetetraacetic acid (EDTA), 1 mM dithiothreitol, 3 μM acetyl-coenzyme A, 10 μM methylmalonyl coenzyme A, 35 μM NADPH.

S1.2. Determination of *α*-glucosidase inhibitory activity

Compounds **1-11** (1.0-1.7 mg) were dissolved in DMSO (100 μL) and then diluted with phosphate buffer (0.1 M, pH 6.8). Sample solution (50 μL, 0.078-78 nM) and 4-nitrophenyl *α*-D-glucopyranoside (pNPG) solution (50 μL, 5 mM) were mixed and incubated in a 96-well microplate at 37 °C for 5 min. *α*-Glucosidase from yeast (50 μL, 0.2 U/mL) was added and incubated at 37 °C for another 30 min. Finally, 50 μL of Na_2CO_3 (1M) was added to terminate the reaction. The absorbance of mixture was measured using a microplate reader at a wavelength 405 nm. The background absorbance (phosphate buffer instead of substrate pNPG) of all samples in no more than 20 μM at 405 nm was little, therefore the inhibitory effect was calculated by the following equation: *α*-glucosidase inhibition (%) = $(A_{\text{control}} - A_{\text{sample}})/A_{\text{control}} \times 100\%$, where A_{control} represented the absorbance of phosphate buffer control without test samples, A_{sample} represented the absorbance of test samples. Acarbose was used as the positive control.

S1.3. Determination of α-amylase inhibitory activity

Phosphate buffer (20 mM, pH 6.9, containing 6 mM NaCl) was used as the solvent in this assay. Sample solution (50 μL, 50-1500 μM) and starch solution (50 μL, 1%, w/v) were mixed and incubated in a 96-well microplate at 37 °C for 10 min. Then, α-amylase solution (50 μL, 0.2 U/mL) was added and the mixture was incubated at 37 °C for an additional 10 min. The reaction was stopped by addition of 3, 5-dinitrosalicylic acid colour reagent (100 μL, 27.6 mM) and the 96-well microplate was immediately heated in 95 °C water bath for 10 min. When the reaction solution cooled to room temperature, all samples were diluted by adding distilled water (50 μL), and then their absorbance was measured using a microplate reader at 540 nm. All samples had little background absorbance (phosphate buffer instead of starch solution) at 540 nm, thus the inhibitory activity was calculated as $(A_{\text{control}} - A_{\text{sample}})/A_{\text{control}} \times 100\%$, in which A_{sample} was the absorbance of the sample and A_{control} was the absorbance of the phosphate buffer control without test samples. Acarbose was used as the positive control.

S1.4. DPPH radical scavenging assay

The DPPH radical scavenging assay was used to evaluate the antioxidant activity of compounds **1-11**. In a 96-well microplate, 100 μL of DPPH solution (200 μM in ethanol) was added to 100 μL sample in ethanol at graded concentrations ranging from 7 to 500 μM. The mixture was incubated in the dark at room temperature for 30 min. The absorbance of the reaction mixture was measured at 517 nm using a microplate reader. The DPPH scavenging activity was calculated by the following formula: DPPH scavenging activity (%) = $(A_{\text{control}} - A_{\text{sample}})/A_{\text{control}} \times 100\%$, where A_{control} was the absorbance of ethanol control without samples, A_{sample} was the absorbance of sample. Ascorbic acid was used as the positive control in the experiment.

S1.5. ABTS radical scavenging assay

The ABTS radical scavenging assay was used also to evaluate the antioxidant activity of compounds **1-11**. The ABTS free radical cation ($ABTS^{\bullet+}$) was manufactured by reacting ABTS stock solution (7 mM) with potassium persulphate

(2.45 mM) in the dark at room temperature for 12-16 h. The $ABTS^{\bullet+}$ solution was diluted with ethanol to an absorbance of 0.7 at 734 nm. Sample solution (100 μL, 2-100 μM in ethanol) was mixed with 150 μL diluted $ABTS^{\bullet+}$ solution. After reaction in the dark at room temperature for 20 min, the absorbance of the reaction mixture at 734 nm was recorded. The $ABTS^{\bullet+}$ scavenging capability was calculated as $(A_{\text{control}} - A_{\text{sample}})/A_{\text{control}} \times 100\%$, in which A_{control} was the absorbance of ethanol control without samples, A_{sample} was the absorbance of sample. Ascorbic acid was used as the positive control.

*S2. ^{1}H, ^{13}C NMR data of **1a**, **4a**, and **7-11***

Compound **1a** (ligurobustoside R): ^{1}H NMR (400 MHz, CD_3OD) δ: aglycone 7.03 (2H, d, J = 8.4 Hz, H-2, 6), 6.65 (2H, d, J = 8.4 Hz, H-3, 5), 2.83 (2H, t, J = 7.2 Hz, H-7), 3.72 (1H, m, H-8a), 3.96 (1H, m, H-8b); Man 4.33 (1H, d, J = 8.0 Hz, H-1'), 3.31 (1H, m, H-2'), 3.54 (1H, m, H-3'), 3.38 (1H, m, H-4'), 3.53 (1H, m, H-5'), 4.33 (1H, dd, J = 11.6, 6.4 Hz, H-6a'), 4.50 (1H, dd, J = 11.6, 2.0 Hz, H-6b'); inner Rha 5.18 (1H, d, J = 2.0 Hz, H-1''), 3.89 (1H, m, H-2''), 3.84 (1H, dd, J = 9.6, 3.2 Hz, H-3''), 3.53 (1H, m, H-4''), 4.10 (1H, m, H-5''), 1.28 (3H, d, J = 6.0 Hz, H-6''); outer Rha 5.19 (1H, d, J = 1.6 Hz, H-1'''), 3.94 (1H, m, H-2'''), 3.60 (1H, dd, J = 9.6, 3.2 Hz, H-3'''), 3.39 (1H, m, H-4'''), 3.70 (1H, m, H-5'''), 1.25 (3H, d, J = 6.4 Hz, H-6'''); Cou 7.41 (2H, d, J = 8.8 Hz, H-2'''', 6''''), 6.79 (2H, d, J = 8.8 Hz, H-3'''', 5''''), 7.62 (1H, d, J = 16.0 Hz, H-7''''), 6.35 (1H, d, J = 16.0 Hz, H-8''''). ^{13}C NMR (100 MHz, CD_3OD) δ: aglycone 130.6 (C-1), 130.9 (C-2,6), 116.1 (C-3,5), 156.7 (C-4), 36.4 (C-7), 72.3 (C-8); Man 104.2 (C-1'), 75.7 (C-2'), 83.6 (C-3'), 70.4 (C-4'), 75.2 (C-5'), 64.4 (C-6'); inner Rha 103.2 (C-1''), 72.8 (C-2''), 73.0 (C-3''), 81.1 (C-4''), 68.4 (C-5''), 18.6 (C-6''); outer Rha 102.4 (C-1'''), 72.3 (C-2'''), 72.3 (C-3'''), 73.8 (C-4'''), 70.4 (C-5'''), 17.8 (C-6'''); Cou 127.5 (C-1''''), 133.7 (C-2'''',6''''), 115.9 (C-3'''',5''''), 160.1 (C-4''''), 145.3 (C-7''''), 116.3 (C-8''''), 168.1 (CO).

Compound **4a** (ligurobustoside S): ^{1}H NMR (400 MHz, CD_3OD) δ: aglycone 7.43 (2H, br. d, J = 7.2 Hz, H-2, 6), 7.35 (2H, br. t, J = 7.2 Hz, H-3, 5), 7.28 (1H, br. d, J = 7.2 Hz, H-4), 4.69 (1H, d, J = 11.6 Hz, H-7a), 4.96 (1H, d, J = 11.6 Hz, H-7b); Man 4.45 (1H, d, J = 8.0 Hz, H-1'), 3.46 (1H, dd, J = 9.2, 8.0 Hz, H-2'), 3.82 (1H, t, J

= 9.2 Hz, H-3'), 4.94 (1H, m, H-4'), 3.54 (1H, m, H-5'), 3.56 (1H, m, H-6a'), 3.64 (1H, m, H-6b'); Rha 5.18 (1H, d, *J* = 1.6 Hz, H-1''), 3.92 (1H, dd, *J* = 3.2, 1.6 Hz, H-2''), 3.58 (1H, m, H-3''), 3.29 (1H, t, *J* = 9.6 Hz, H-4''), 3.56 (1H, m, H-5''), 1.08 (3H, d, *J* = 6.0 Hz, H-6''); Cou 7.47 (2H, d, *J* = 8.4 Hz, H-2''', 6'''), 6.81 (2H, d, *J* = 8.4 Hz, H-3''', 5'''), 7.67 (1H, d, *J* = 16.0 Hz, H-7'''), 6.35 (1H, d, *J* = 16.0 Hz, H-8'''). ^{13}C NMR (100 MHz, CD_3OD) δ: aglycone 139.0 (C-1), 129.3 (C-2,6), 129.1 (C-3,5), 128.7 (C-4), 72.0 (C-7); Man 103.2 (C-1'), 76.2 (C-2'), 81.7 (C-3'), 70.6 (C-4'), 76.1 (C-5'), 62.4 (C-6'); Rha 103.1 (C-1''), 72.3 (C-2''), 72.0 (C-3''), 73.8 (C-4''), 70.4 (C-5''), 18.4 (C-6''); Cou 127.1 (C-1'''), 131.3 (C-2''',6'''), 116.8 (C-3''',5'''), 161.4 (C-4'''), 147.6 (C-7'''), 114.8 (C-8'''), 168.3 (CO).

Compound **7** (ligupurpuroside B): white amorphous powder. ^{1}H NMR (400 MHz, CD_3OD) δ: aglycone 7.07 (2H, d, *J* = 8.4 Hz, H-2, 6), 6.71 (2H, d, *J* = 8.4 Hz, H-3, 5), 2.84 (2H, t, *J* = 7.6 Hz, H-7), 3.90 (1H, m, H-8a), 4.06 (1H, m, H-8b); Glc 4.38 (1H, d, *J* = 7.6 Hz, H-1'), 3.37 (1H, m, H-2'), 3.77 (1H, t, *J* = 9.6 Hz, H-3'), 4.91 (1H, t, *J* = 9.6 Hz, H-4'), 3.46 (1H, m, H-5'), 3.50 (1H, m, H-6a'), 3.57 (1H, m, H-6b'); inner Rha 5.19 (1H, d, *J* = 2.0 Hz, H-1''), 3.86 (1H, dd, *J* = 3.2, 2.0 Hz, H-2''), 3.68 (1H, dd, *J* = 9.6, 3.2 Hz, H-3''), 3.39 (1H, m, H-4''), 3.59 (1H, m, H-5''), 1.09 (3H, d, *J* = 6.0 Hz, H-6''); outer Rha 5.04 (1H, d, *J* = 2.0 Hz, H-1'''), 3.90 (1H, dd, *J* = 3.2, 2.0 Hz, H-2'''), 3.51 (1H, m, H-3'''), 3.32 (1H, m, H-4'''), 3.46 (1H, m, H-5'''), 1.04 (3H, d, *J* = 6.0 Hz, H-6'''); Cou 7.48 (2H, d, *J* = 8.4 Hz, H-2'''', 6''''), 6.83 (2H, d, *J* = 8.4 Hz, H-3'''', 5''''), 7.66 (1H, d, *J* = 16.0 Hz, H-7''''), 6.33 (1H, d, *J* = 16.0 Hz, H-8''''). ^{13}C NMR (100 MHz, CD_3OD) δ: aglycone 130.7 (C-1), 130.9 (C-2,6), 116.2 (C-3,5), 156.8 (C-4), 36.3 (C-7), 72.4 (C-8); Glc 104.2 (C-1'), 76.2 (C-2'), 81.6 (C-3'), 70.5 (C-4'), 75.8 (C-5'), 62.4 (C-6'); inner Rha 102.7 (C-1''), 72.7 (C-2''), 72.9 (C-3''), 81.6 (C-4''), 68.9 (C-5''), 19.2 (C-6''); outer Rha 103.5 (C-1'''), 72.3 (C-2'''), 72.3 (C-3'''), 73.8 (C-4'''), 70.3 (C-5'''), 17.7 (C-6'''); Cou 127.0 (C-1''''), 131.4 (C-2'''',6''''), 117.1 (C-3'''',5''''), 161.3 (C-4''''), 147.6 (C-7''''), 114.7 (C-8''''), 168.1 (CO).

Compound **8** (*cis*-ligupurpuroside B): white amorphous powder. ^{1}H NMR (400 MHz, CD_3OD) δ: aglycone 7.07 (2H, d, *J* = 8.4 Hz, H-2, 6), 6.70 (2H, d, *J* = 8.4 Hz, H-3, 5), 2.84 (2H, t, *J* = 7.6 Hz, H-7), 3.70 (1H, m, H-8a), 4.03 (1H, m, H-8b); Glc 4.36 (1H, d, *J* = 8.0 Hz, H-1'), 3.37 (1H, m, H-2'), 3.77 (1H, t, *J* = 9.6 Hz, H-3'), 4.88 (1H, t, *J* = 9.6 Hz, H-4'), 3.46 (1H, m, H-5'), 3.50 (1H, m, H-6a'), 3.57 (1H, m, H-6b'); inner Rha 5.29 (1H, d, *J* = 1.6 Hz, H-1''), 3.82 (1H, dd, *J* = 3.2, 1.6 Hz, H-2''), 3.68 (1H, dd, *J* = 9.6, 3.2 Hz, H-3''), 3.45 (1H, m, H-4''), 3.60 (1H, m, H-5''), 1.22 (3H, d, *J* = 6.0 Hz, H-6''); outer Rha 5.13 (1H, d, *J* = 1.6 Hz, H-1'''), 3.82 (1H, dd, *J* = 3.2, 1.6 Hz, H-2'''), 3.51 (1H, m, H-3'''), 3.34 (1H, m, H-4'''),

3.46 (1H, m, H-5'''), 1.21 (3H, d, *J* = 6.4 Hz, H-6'''); Cou 7.72 (2H, d, *J* = 8.8 Hz, H-2'''', 6''''), 6.77 (2H, d, *J* = 8.8 Hz, H-3'''', 5''''), 6.99 (1H, d, *J* = 12.8 Hz, H-7''''), 5.76 (1H, d, *J* = 12.8 Hz, H-8''''). ^{13}C NMR (100 MHz, CD_3OD) δ: aglycone 130.7 (C-1), 130.9 (C-2,6), 116.1 (C-3,5), 156.8 (C-4), 36.3 (C-7), 72.4 (C-8); Glc 104.2 (C-1'), 76.4 (C-2'), 79.6 (C-3'), 70.4 (C-4'), 75.9 (C-5'), 62.4 (C-6'); inner Rha 101.9 (C-1''), 72.9 (C-2''), 73.0 (C-3''), 80.6 (C-4''), 68.6 (C-5''), 18.9 (C-6''); outer Rha 103.2 (C-1'''), 72.3 (C-2'''), 72.3 (C-3'''), 73.9 (C-4'''), 70.3 (C-5'''), 17.8 (C-6'''); Cou 127.5 (C-1''''), 134.3 (C-2'''',6''''), 115.9 (C-3'''',5''''), 160.3 (C-4''''), 147.4 (C-7''''), 115.8 (C-8''''), 166.9 (CO).

Compound **9** (ligurobustoside N): yellow amorphous powder. ^{1}H NMR (400 MHz, CD_3OD) δ: aglycone 7.07 (2H, d, *J* = 8.4 Hz, H-2, 6), 6.70 (2H, d, *J* = 8.4 Hz, H-3, 5), 2.84 (2H, dt, *J* = 7.2, 2.0 Hz, H-7), 3.68 (1H, m, H-8a), 3.90 (1H, m, H-8b); Glc 4.38 (1H, d, *J* = 8.0 Hz, H-1'), 3.37 (1H, m, H-2'), 3.77 (1H, t, *J* = 9.6 Hz, H-3'), 4.93 (1H, t, *J* = 9.6 Hz, H-4'), 3.46 (1H, m, H-5'), 3.50 (1H, m, H-6a'), 3.57 (1H, m, H-6b'); inner Rha 5.20 (1H, d, *J* = 2.0 Hz, H-1''), 3.86 (1H, dd, *J* = 3.2, 2.0 Hz, H-2''), 3.68 (1H, dd, *J* = 9.6, 3.2 Hz, H-3''), 3.39 (1H, m, H-4''), 3.59 (1H, m, H-5''), 1.09 (3H, d, *J* = 6.4 Hz, H-6''); outer Rha 5.05 (1H, d, *J* = 1.6 Hz, H-1'''), 3.90 (1H, dd, *J* = 3.2, 1.6 Hz, H-2'''), 3.51 (1H, m, H-3'''), 3.32 (1H, m, H-4'''), 3.46 (1H, m, H-5'''), 1.06 (3H, d, *J* = 6.4 Hz, H-6'''); Caff 7.07 (1H, d, *J* = 2.0 Hz, H-2''''), 6.79 (1H, d, *J* = 8.0 Hz, H-5''''), 6.97 (1H, dd, *J* = 8.0, 2.0 Hz, H-6''''), 7.60 (1H, d, *J* = 16.0 Hz, H-7''''), 6.26 (1H, d, *J* = 16.0 Hz, H-8''''). ^{13}C NMR (100 MHz, CD_3OD) δ: aglycone 130.7 (C-1), 130.9 (C-2,6), 116.1 (C-3,5), 156.8 (C-4), 36.4 (C-7), 72.3 (C-8); Glc 104.2 (C-1'), 76.2 (C-2'), 81.5 (C-3'), 70.3 (C-4'), 76.0 (C-5'), 62.3 (C-6'); inner Rha 103.4 (C-1''), 73.8 (C-2''), 70.4 (C-3''), 81.5 (C-4''), 68.9 (C-5''), 19.2 (C-6''); outer Rha 102.7 (C-1'''), 72.4 (C-2'''), 72.3 (C-3'''), 73.8 (C-4'''), 68.9 (C-5'''), 17.7 (C-6'''); Caff 127.5 (C-1''''), 115.3 (C-2''''), 146.8 (C-3''''), 149.9 (C-4''''), 116.7 (C-5''''), 123.4 (C-6''''), 148.0 (C-7''''), 114.7 (C-8''''), 168.1 (CO).

Compound **10** (osmanthuside D): white amorphous powder. ^{1}H NMR (400 MHz, CD_3OD) δ: aglycone 7.07 (2H, d, *J* = 8.4 Hz, H-2, 6), 6.70 (2H, d, *J* = 8.4 Hz, H-3, 5), 2.85 (2H, dt, *J* = 7.6, 2.0 Hz, H-7), 3.75 (1H, m, H-8a), 4.05 (1H, m, H-8b); Glc 4.35 (1H, d, *J* = 8.0 Hz, H-1'), 3.52 (1H, m, H-2'), 3.82 (1H, t, *J* = 9.2 Hz, H-3'), 4.89 (1H, m, H-4'), 3.58 (1H, m, H-5'), 3.56 (1H, m, H-6a'), 3.64 (1H, m, H-6b'); Rha 5.16 (1H, d, *J* = 1.6 Hz, H-1''), 3.92 (1H, dd, *J* = 3.2, 1.6 Hz, H-2''), 3.60 (1H, m, H-3''), 3.29 (1H, m, H-4''), 3.58 (1H, m, H-5''), 1.16 (3H, d, *J* = 6.4 Hz, H-6''); Cou 7.72 (2H, d, *J* = 8.8 Hz, H-2''', 6'''), 6.76 (2H, d, *J* = 8.8 Hz, H-3''', 5'''), 6.95 (1H, d, *J* = 12.8 Hz, H-7'''), 5.79 (1H, d, *J* = 12.8 Hz, H-8'''). ^{13}C NMR

(100 MHz, CD_3OD) δ: aglycone 130.7 (C-1), 131.3 (C-2, 6), 116.1 (C-3, 5), 156.8 (C-4), 36.4 (C-7), 72.1 (C-8); Glc 104.2 (C-1'), 76.0 (C-2'), 81.8 (C-3'), 70.5 (C-4'), 76.1 (C-5'), 62.4 (C-6'); Rha 103.1 (C-1''), 72.3 (C-2''), 72.2 (C-3''), 73.9 (C-4''), 70.4 (C-5''), 18.2 (C-6''); Cou 127.5 (C-1'''), 134.3 (C-2''', 6'''), 115.8 (C-3''', 5'''), 160.4 (C-4'''), 147.3 (C-7'''), 115.9 (C-8'''), 166.9 (CO).

Compound **11** [(*Z*)-osmanthuside B_6]: white amorphous powder. ^{1}H NMR (600 MHz, CD_3OD) δ: aglycone 7.02 (2H, d, J = 8.4 Hz, H-2, 6), 6.67 (2H, d, J = 8.4 Hz, H-3, 5), 2.83 (2H, t, J = 7.8 Hz, H-7), 3.70 (1H, m, H-8a), 3.93 (1H, m, H-8b); Glc 4.28 (1H, d, J = 7.8 Hz, H-1'), 3.33 (1H, m, H-2'), 3.53 (1H, t, J = 9.0 Hz, H-3'), 3.40 (1H, m, H-4'), 3.55 (1H, m, H-5'), 4.31 (1H, dd, J = 12.0, 6.0 Hz, H-6a'), 4.48 (1H, dd, J = 12.0, 2.4 Hz, H-6b'); Rha 5.16 (1H, d, J = 1.8 Hz, H-1''), 3.94 (1H, m, H-2''), 3.70 (1H, m, H-3''), 3.40 (1H, t, J = 9.6 Hz, H-4''), 4.00 (1H, dd, J = 9.6, 6.6 Hz, H-5''), 1.24 (3H, d, J = 6.6 Hz, H-6''); Cou 7.63 (2H, d, J = 8.4 Hz, H-2''', 6'''), 6.74 (2H, d, J = 8.4 Hz, H-3''', 5'''), 6.87 (1H, d, J = 12.6 Hz, H-7'''), 5.79 (1H, d, J = 12.6 Hz, H-8'''). ^{13}C NMR (100 MHz, CD_3OD) δ: aglycone 130.6 (C-1), 131.2 (C-2, 6), 116.3 (C-3, 5), 156.8 (C-4), 36.4 (C-7), 72.3 (C-8); Glc 104.3 (C-1'), 75.6 (C-2'), 83.9 (C-3'), 70.4 (C-4'), 75.4 (C-5'), 64.4 (C-6'); Rha 102.7 (C-1''), 72.3 (C-2''), 72.2 (C-3''), 74.0 (C-4''), 70.0 (C-5''), 17.9 (C-6''); Cou 127.6 (C-1'''), 133.7 (C-2''', 6'''), 116.1 (C-3''', 5'''), 160.1 (C-4'''), 145.3 (C-7'''), 115.9 (C-8'''), 168.1 (CO).
